# Knockout of *secondary alcohol dehydrogenase* in *Nocardia cholesterolicum* NRRL 5767 by CRISPR/Cas9 genome editing technology

**DOI:** 10.1371/journal.pone.0230915

**Published:** 2020-03-27

**Authors:** Jenq-Kuen Huang, Kadidia Samassekou, Hekmat B. Alhmadi, David R. VanDerway, Joshua D. Diaz, Jacob A. Seiver, Shawn W. McClenahan, Scott M. Holt, Lisa Wen

**Affiliations:** 1 Department of Chemistry, Western Illinois University, Macomb, IL, United States of America; 2 Department of Biological Sciences, Western Illinois University, Macomb, IL, United States of America; Purdue University, UNITED STATES

## Abstract

*Nocardia cholesterolicum* NRRL 5767 is well-known for its ability to convert oleic acid to 10-hydroxystearic acid (~88%, w/w) and 10-ketostearic acid (~11%, w/w). Conversion of oleic acid to 10-hydroxystearic acid and then to 10-ketostearic acid has been proposed to be catalyzed by oleate hydratase and secondary alcohol dehydrogenase, respectively. Hydroxy fatty acids are value-added with many industrial applications. The objective of this study was to improve the *Nocardia cholesterolicum* NRRL5767 strain by CRISPR/Cas9 genome editing technology to knockout the *secondary alcohol dehydrogenase* gene, thus blocking the conversion of 10-hydroxystearic acid to 10-ketostearic acid. The improved strain would produce 10-hydroxystearic acid solely from oleic acid. Such improvement would enhance the production of 10-hydroxystearic acid by eliminating downstream separation of 10-hydroxystearic acid from 10-ketostearic acid. Here, we report: (1) Molecular cloning and characterization of two functional recombinant oleate hydratase isozymes and a functional recombinant secondary alcohol dehydrogenase from *Nocardia cholesterolicum* NRRL5767. Existence of two oleate hydratase isozymes may explain the high conversion yield of 10-hydroxystearic acid from oleic acid. (2) Construction of a CRISPR/Cas9/sgRNA chimeric plasmid that specifically targeted the *secondary alcohol dehydrogenase* gene by Golden Gate Assembly. (3) Transformation of the chimeric plasmid into *Nocardia cholesterolicum* NRRL 5767 by electroporation and screening of secondary alcohol dehydrogenase knockout mutants. Two mutants were validated by their lack of secondary alcohol dehydrogenase activity at the protein level and mutation at the targeted 5’ coding region and the 5’ upstream at the DNA level. The knockout mutants offer improvements by converting added oleic acid to solely 10-hydroxystearic acid, thus eliminating downstream separation of 10-hydroxystearic acid from 10-ketostearic acid. To the best of our knowledge, we report the first successful knockout of a target gene in the *Nocardia* species using CRISPR/Cas9/sgRNA-mediated genome editing technology.

## 1. Introduction

Hydroxy fatty acids (HFAs) have potential industrial applications as lubricants, waxes, resins, nylons, plastics, cosmetics, additives in coating and paintings, flavors, antimicrobial agent, or precursors for lactones and dicarboxylic acids [1–5]. Unsaturated fatty acids in plant oils can be converted to HFAs chemically or enzymatically [6–10]. There are numerous reports on microbial transformation of edible oils or free fatty acids to value-added HFAs. They occurred via distinct processes/enzymes such as oleate hydratase (Ohase), P450 monooxygenase, lipoxygenase, or hydroxylase [1, 9, 11–19].

In the literature, three microbes have been reported to produce high yields of 10-hydroxysteatic acid (10-HSA) from oleic acid (OA) and 10-hydroxy-12(Z)octadecenoic acid (10OH-12OD) from linoleic acid (LA) [12, 17, 20]. Among them, *Nocardia cholesterolicum* NRRL 6767 (*N*. *cholesterolicum* NRRL5767) is the interest of our on-going research because it produces high yields and it has been proven to be a stable microbe with industrial applications [21–23]. It has been reported that resting cells of *N*. *cholesterolicum* NRRL 5767 are able to convert added OA to ~88% of 10-HSA (w/w) and 11% of 10-ketostearic acid (10-KSA, w/w) [12]. The production of both 10-HSA and 10-KSA complicates downstream separation and purification of 10-HSA. The enzymes involved in OA metabolism to 10-HSA and 10-KSA have been established [24–31], Ohase converts OA to 10-HSA, which is subsequently converted to 10-KSA by a secondary alcohol dehydrogenase (2^o^-ADH).

Ohase catalyzes the addition of a H_2_O molecule to a cis C = C in OA to form 10-HSA [1, 9]. The hydration reaction is regioselective, stereoselective, and functional group selective [11, 19, 24, 32]. Ohase was first purified and then the gene was cloned from *Elizabethkingia meningoseptica* [32]. This work assisted in the functional identification of the myosin cross-reactive antigen (MCRA) protein family as flavin adenine dinucleotide (FAD)-containing fatty acid double bond hydratases due to high degree of sequence homology [32, 33]. Thereafter, numerous articles regarding isolation of Ohases, cloning and characterization of functional recombinant Ohases from various microorganisms were reported [34–42].

X-ray crystal structures of Ohases have been determined from four sources [43–46]. The enzymes were reported to possess a Rossmann fold or mononucleotide-binding fold, a super-secondary structure identified by a repeating short amino acid residue motif, Gly-x-Gly-x-x-Gly (where x denotes any amino acid residue) [33, 35, 47, 48]. This motif is composed of alternating β strands and α helices, βαβ, which are in contact with the adenosine diphosphate region of FAD cofactor. X-ray crystal structures of FAD bound Ohase from *Elizabethkingia meningoseptica* [44] provided an understanding of the role of FAD and the involvement of conserved amino acid residues in the regioselective and stereoselective hydration. The active site amino acid residues E_122_ and Y_241_ act as acid-base catalysts in the hydration of the cis C = C bond; Y_241_ protonates the double bond and E_122_ activates a H_2_O molecule for the *re*-side attack on the partially charged double bond. The FAD plays a dual role in the proper organization of the active site and the stabilization of the partial positive charge in the putative transition state of the hydration reaction [44]. Although many Ohases require FAD for activity [33, 35, 36, 49], some Ohases do not depend on FAD [45, 50], including the two Ohase isozymes (NcOhy1 and NcOhy2) from this study.

Secondary alcohol dehydrogenases (2^o^-ADHs) catalyze the conversion of secondary alcohols of short to medium straight-chain alkanes to the corresponding ketone derivatives in oxidation reactions at pH of 8–9 in the presence of NAD^+^ (or NADP^+^). The same enzymes also catalyze reverse reduction reactions by converting the ketone derivatives to the original substrates at pH 4–5 in the presence of NADH (or NADPH) [51, 52]. In most cases, the substrates contained a hydroxyl group at carbon-2 of straight-chain alkanes ranging from 2 to 14 carbons [53].

A 2^o^-ADH with unique substrate specificity that preferentially oxidizes long-chain hydroxy fatty acids including 10-HSA and 12-hydroxystearic (12-HSA) to their corresponding keto fatty acid derivatives was first isolated from *Pseudomonas sp*. NRRL B3266 [26]. A 2^o^-ADH with similar substrate specificity was isolated from *Micrococcus luteus* WIUJH20 (*M*. *luteus* WIUJH20) by our research team. The gene encoding this 2^o^-ADH has been cloned and a functional recombinant 2^o^-ADH purified and characterized [27] (Accession E2D104). This sequence was used to identify a *2*^*o*^*-ADH* gene in *N*. *cholesterolicum* NRRL 5767 in this study.

Clustered Regularly Interspaced Short Palindromic Repeats (CRISPR) and their CRISPR-associated (Cas) proteins constitute the CRISPR/Cas immune system which plays a key role in adaptive immunity against invasive viruses and plasmids in bacteria and archaea [54, 55]. The CRISPR/Cas9 genome editing system is guided by “protospacer” sequences that are transcribed into short RNA sequences (“CRISPR RNAs” or “crRNAs”) capable of guiding the system to matching sequences of DNA target(s). Once the target DNA is found, Cas9 (an endonuclease produced by the CRISPR system) binds to the DNA and cuts it creating double-strand breaks (DSBs), shutting the targeted gene off. The technology has provided researchers with the ability to create DSBs at any desired position in a genome. In prokaryotes, the non-homologous end-joining (NHEJ) pathway is one of the major mechanisms for repairing DSBs that occur in genomic DNA [56]. The NHEJ pathway is intrinsically error-prone, typically resulting in small insertions and/or deletions (indels) at the site of the break. The indels are likely to cause a frameshift mutation; can knockout the function of the target gene due to the production of truncated polypeptides.

Despite RNA-guided CRISPR/Cas9 genome editing technology being successfully employed in eukaryotes, only in a few cases has it been successfully employed in bacteria using dual-RNA-guided CRISPR/Cas9 [57, 58]. Recently, the engineered CRISPR/Cas9 system has been successfully utilized for genome editing of *Streptomyces* or *Actinomyces* [59–61], *Enterobacteriaceae* a family of *Tatumella citrea* [62], and *Clostridium beijerinckii* [63, 64].

The goal of this research is to convert OA and LA in acidulated soapstock to HFAs in high yields by biotechnology. Renewable corn and soybean are the main agricultural commodities of Illinois and in the USA. Acidulated soapstock, a byproduct of corn and soybean edible oil refinery, is plentiful and relatively inexpensive. It contains ~16% free fatty acids, existing primarily as OA and LA [65], and has historically been sold to the chicken feed industry at a low cash value. Using a cheaper source of OA and LA to produce value-added HFAs would have significant economic advantages.

The objective of our research was to improve the *N*. *cholesterolicum* NRRL5767 strain by CRISPR/Cas9 genome editing technology to knockout the 2^o^-*ADH* gene so that a new metabolically engineered *N*. *cholesterolicum* NRRL5767 strain would produce 10-HSA solely from OA substrate. In order to engineer the *N*. *cholesterolicum* NRRL 5767 strain, the enzymes involved in OA metabolism, namely Ohase and 2^o^-ADH, must be cloned and characterized. Through the first draft genome sequence of *N*. *cholesterolicum* NRRL 5767 and genome annotation, we identified two *Ohases* and one *2*^*o*^*-ADH* genes from *N*. *cholesterolicum* NRRL5767. Each gene was amplified from the genomic DNA by PCR and cloned into an expression vector and functional enzymes overexpressed, purified, and characterized.

Next, a single-guide RNA (sgRNA) that specifically targeted the 5’coding sequence of the *2*^*o*^*-ADH* gene was designed and a CRISPR/Cas9/sgRNA chimeric plasmid was constructed by Golden Gate Assembly. The chimeric plasmid construct was then transformed into *N*. *cholesterolicum* NRRL 5767 by electroporation for sgRNA expression and gene editing. Transformants were screened by antibiotic resistance and the ability to convert OA solely to 10-HSA (without 10-KSA).

We have successfully isolated two knockout mutants that have been validated at both protein and DNA levels. At the protein level, the mutants transform added OA to 10-HSA solely with no detectable 10-KSA demonstrating the lack of 2^o^-ADH activity. At the DNA level, mutation was shown to occur on-target at the 5’ coding sequence and extend to its upstream location of the *2*^*o*^*-ADH* gene. This is the first report of successful genome editing in *Nocardia* species using CRISPR/Cas9 genome editing technology.

## 2. Materials and methods

Unless otherwise mentioned, all materials were obtained from vendors or research institutes in the United States. All chemicals used were ACS grade.

### 2.1. Microorganisms and plasmids

*N*. *cholesterolicum* NRRL 5767 was a gift from USDA ARS Culture and Patent Culture Collections, Peoria, Illinois. JM109 and NEB 5-α competent cells were from New England Biolabs. BL21(DE3)pLysS was from Invitrogen. Protein expression vectors pET-15b and pET-28a were from Novagen. pCRISPomyces-2 was a gift from Huimin Zhao [59] (Addgene plasmid # 61737; http://n2t.net/addgene:61737). Various recombinant clones obtained throughout this research were suspended in 20% glycerol for long term storage at -70°C.

### 2.2. Isolation of genomic DNA from *N*. *cholesterolicum* NRRL5767

Genomic DNA was isolated from *N*. *cholesterolicum* NRRL5767 according to the procedure of Flamm *et al* [66] with minor modifications. Two mL of freshly cultured cells were centrifuged. The cell pellet was suspended in 1 mL of 10 mM sodium phosphate, pH 7.0 containing 20% sucrose and 2.5 mg lysozyme and incubated at 37°C overnight. The following day, 9 mL of lysis buffer (10 mM Tris-HCl, pH 8.0, 1 mM EDTA, 1% SDS, and a pinch of proteinase K) was added and incubated at 37°C for an hour. The cell lysate was extracted with phenol/chloroform and the DNA was precipitated from the aqueous layer with sodium acetate and ethanol by gently inverting the tube several times. The DNA was spooled out with a glass rod, washed with 75% ethanol, air-dried, and dissolved in 400 μLT_10_E_1_. The purity and quality of the DNA was examined by the ratio of the absorbance at 260 nm and 280 nm (> 1.8) and by agarose gel electrophoresis. The genomic DNA was sent to the Roy J. Carver Biotechnology Center/W.M. Keck Center Carl R. Woese Institute for Genomic Biology, University of Illinois at Urbana-Champaign for the first draft genomic DNA sequencing and genome annotation.

### 2.3. PCR amplification and cloning of putative *Ohase* genes

To find the putative *Ohase* gene in the *N*. *cholesterolicum* NRRL5767 genomic data, a homology search with the amino acid sequence of Ohase from *Elizabethkingia meningoseptica* (Accession GQ144652.1) was performed using Protein BLAST. Two annotated *Ohase* genes, PROKKA_00231 and PROKKA_02988, were identified on the genome of *N*. *cholesterolicum* NRRL5767. Each putative Ohase gene, designated *NcOhy1* (PROKKA*_*00231) and *NcOhy2* (PROKKA*_*02988), was amplified from the genomic DNA by PCR using a pair of primers flanking each target. The nucleotide sequences of the primer pairs are shown in Table 1. To facilitate cloning, HindIII restriction enzyme recognition sequence (AAGCTT, underlined) was appended to the 5’ ends of both forward and reverse primers. Additional 6 nucleotides, CGATAT was appended to the 5’ end of each primer to facilitate subsequent cleavage of the PCR product by HindIII. In the forward primer, two additional nucleotides, GC, were placed right after the HindIII site to edit the reading frame in order to ensure the expression of desired recombinant protein.

**Table 1:** Primers for amplification of the putative *Ohase* genes and *2*^*o*^*-ADH* gene.

Primer pair for *NcOhy1* (PROKKA_00231)	
Forward with HindIII site	CGATATAAGCTT**GCATGTATTACAGCAGTGGAAACTACGAAGCGTTC**
Reverse with HindIII site	CGATATAAGCTT**TCATTCTCGGGGGAGAATGTGGTATTGG**
Primer pair for *NcOhy2* (PROKKA_02988)	
Forward with HindIII site	CGATATAAGCTT**GCATGTCTTCCAATCTGTCTCACAAGGCC**
Reverse with HindIII site	CGATATAAGCTT**TCAGCGGAACATCGTCACTGC**
Primer pairs for *Nc2*^*o*^*-ADH* (PROKKA_03439)	
Forward	**ATGACTGAACTGAAGCAGATCACCG**
Reverse	**TCAGCCTTTGTAGTTGTAGAAGCCC**
Forward with XhoI site	ATGCGACTCGAG**ATGACTGAACTGAAGCAGATCACCG**
Reverse with XhoI site	ATGCGACTCGAG**TCAGCCTTTGTAGTTGTAGAAGCCC**

Bold-typed letters indicate the coding sequence. Cloning sites are underlined. The additional 6 nucleotides at the 5’ end was to facilitate digestion of the PCR products by the restriction enzyme.

Each set of PCR (50 μL) consisted of 0.2 μM each of the forward and reverse primer, 1x FideliTaq PCR Master Mix (United States Biochemicals), 100 ng genomic DNA, and 2% DMSO. The PCR program consisted of pre-denaturation at 95°C for 2 min; 25 cycles of denaturation (95°C, 30 sec), annealing (54.8°C, 30 sec), and primer extension (68°C, 1.5 min); and a final 5 min primer extension at 68°C. An aliquot of each PCR product was analyzed on a 1% agarose gel. Once the desired band was observed, the remaining PCR product was extracted with phenol/chloroform, chloroform/isoamyl alcohol, precipitated with ethanol [67]. Each PCR product of *NcOhy1* or *NcOhy2* was digested with HindIII and the fragments separated by agarose gel. The desired bands were purified from the gel, cloned into pET-28a (digested with HindIII and dephosphorylated), transformed into JM109 competent cells, and plated on agar plates containing kanamycin [67]. Transformants were checked for the presence of chimeric plasmid with DNA insert in the correct orientation by colony PCR using universal T7 primer and a gene specific reverse primer. Conditions for colony PCR were similar except that 2x PCR master mix was from Promega, the extension temperature was at 72°C, and 35 cycles were carried out. The desired PCR product should only be found in clones harboring an insert in the correct orientation. Chimeric plasmids were then isolated from a few of these clones by alkaline lysis and further purified using QIAprep 2.0 spin columns according to the manufacturer’s protocol. The samples were dried by SpeedVac and sent to Laragen for DNA sequencing of both strands using Sanger’s chain termination method. The identities of the clones were verified by comparing the DNA sequence and the inferred amino acid sequence with the annotated gene sequences from the *N*. *cholesterolicum* NRRL5767 genomic data. The correct recombinant plasmids were each transformed into BL21(DE3)pLysS host cells for protein expression.

### 2.4. PCR amplification and cloning of putative *2*^*o*^*-ADH* gene

To find the putative *2*^*o*^*-ADH* gene in the *N*. *cholesterolicum* NRRL5767 genomic data, homology search with amino acid sequence of 2^o^-ADH from *M*. *luteus* WIUJH20 [27] (Accession E2D104) was performed using Protein BLAST. The best hit from this search was an annotated 3-hydroxybutyryl-CoA dehydrogenase (refer to as Nc2^o^-ADH in this paper). Using this sequence information, two primer pairs were designed for amplification of the coding region by PCR. The first pair of the forward and reverse primers contains only the coding sequence (Table 1, bold-typed). The second primer pair contains XhoI recognition sequence (underlined) and 6 additional nucleotides at the 5’ end (Table 1).

The primer pair that contains only the coding sequence was used to amplify the target gene from the genomic DNA of *N*. *cholesterolicum* NRRL5767 to reduce the possibility of off-target amplification. The PCR was carried out in the same manner as for the amplification of Ohase genes except the annealing temperature was changed to 53.7°C. Once the desired PCR band was observed, the purified PCR product was then used as template for amplification using gene specific primers containing XhoI recognition sequence and additional 6 nucleotides (Table 1). The PCR products were extracted, digested with XhoI, and separated on a 1% agarose gel. The desired bands were gel purified, cloned into pET15b (digested with XhoI and dephosphorylated), and transformed into JM109 competent cells. The transformants were selected by ampicillin resistance followed by Quick Screening [68]. The orientation of the insert in the selected colonies was checked by colony PCR as described before. Subsequently, the plasmids were purified and sent to Laragen Inc. for DNA sequencing of both strands. The identity of the clones was verified by comparing the DNA sequence and the inferred amino acid sequence with the annotated gene. The correct recombinant plasmid was then transformed into BL21(DE3)pLysS host cells for protein expression.

### 2.5. Overexpression and purification of recombinant NcOhy1, NcOhy2, and Nc2^o^-ADH proteins

The transformants of BL21(DE3)pLysS that harbored the chimeric plasmid encoding NcOhy1, NcOhy2, or Nc2^o^-ADH were induced for protein expression in the presence of IPTG [69]. The Ohase clones were cultured in terrific broth containing 25 μg/ml kanamycin and 20 μg/ml chloramphenicol. Induction of the 2^o^-ADH recombinant protein was carried out in the same manner except that 100 μg/mL of ampicillin was used instead of kanamycin.

Each of the recombinant proteins was purified from cell-free extract using Ni^2+^-NTA HisTrap^TM^ FF affinity column (5 mL prepacked) (GE Healthcare) according to the manufacturer’s instructions. After washing, the recombinant protein was eluted with elution buffer and ~1 mL fractions were collected. Protein concentration in each fraction was determined by Bradford assay and fractions with high protein contents were pooled. The pooled fractions were dialyzed overnight at 4°C against several changes of dialysis buffer (50 mM Tris-HCl/150 mM NaCl/0.5 mM β-ME, pH 8.0). Protein purification progress and purity were checked by SDS-PAGE.

### 2.6. Ohase activity assay

To determine if each of the overexpressed recombinant proteins (NcOhy1 and NcOhy2) possessed Ohase activity, an assay was carried out according to Koritala *et al* [12, 13] which is based on the ability of the enzyme to convert OA to 10-HSA or LA to 10OH-12OD. Each Ni-affinity purified recombinant protein was used to set up bioconversion reactions. Four mg of purified NcOhy1 or NcOhy2 was reacted with 15 μL OA or LA. The enzymes were also tested for pH dependence at varying pH: 50 mM sodium acetate, pH 4.0; 50 mM MES, pH 6.5; 50 mM Tris-HCl, pH 8.0; or 50 mM sodium borate, pH 9.5 using OA as the substrate. Each reaction was carried out at 37°C, 200 rpm for 8 hr. The reaction products were extracted with ethyl ether [12, 13], analyzed by TLC, and structure confirmed by GC-MS.

#### 2.6.1. Detection of the conversion products by TLC analysis

The reaction products were acidified and extracted with ethyl ether three times [12, 13]. The combined ether extract was washed once with equal volume of deionized H_2_O followed by centrifugation. The ether layer was collected, dried with N_2_ gas, and dissolved in 300 μL ethyl ether. Each extracted sample (40 μL) was applied onto a TLC plate and developed using a solvent consisted of n-hexane/ethyl ether/acetic acid (79/14/7 = v/v/v). A fatty acid standard (a mixture of OA, 10-KSA, and 10-HSA) was also applied to the same plate to serve as references. After developing, the plate was briefly soaked in 8N H_2_SO_4_ followed by heating at about 500°C using a heat gun to char the organic compounds. The conversion products were tentatively identified by comparing their R_f_ values with that of authentic fatty acids. They were further confirmed by GC-MS.

#### 2.6.2. Identification of conversion products by GC-MS

After separation of the conversion products by TLC, the plate by-passed the soaking and heating steps. Instead, it was air-dried in a fume hood and the plate was viewed against fluorescent light to locate and circle 10-HSA or 10-KSA band on the plate. The desired band was then etched out, extracted with ethyl ether, and dried with N_2_ gas. The product was silylated with Tri-Sil^®^ TBT reagent, heated at 75°C for 30 min according to the manufacturer’s instructions (Thermo Scientific Pierce). The GC-MS analysis was done using the services provided by The School of Chemical Sciences Mass Spectrometry Laboratory at University of Illinois at Urbana-Champaign. Briefly, Waters GCT Premier-TOF Mass Spectrometer was used and the conditions were: injecting port at 280°C, 1μL sample was injected into a DB-5 fused silica capillary column (30 m x 0.25 mm, film thickness 0.25 μm). Initial column temperature was at 120°C for 1 min, ramping at 10°C/min for 16 min, and at 280°C for 13 min. Flow rate of helium carrier gas was 60 mL/min, split ratio was 60. Peaks were identified by comparison to the fatty acid standard.

#### 2.6.3. Quantification of conversion products by GC

The conversion reaction conditions were set up as follows: 1.68 μM of NcOhy1 or 6.7 μM of NcOhy2 was mixed with 300 μM of OA, 0.02% Brij 56, and 50 mM sodium phosphate, pH 6.7 in a total reaction volume of 120 μL. The reaction was carried out at 37°C for 5 min. Prior to extraction with ethyl ether, each sample was spiked with heptadecanoic acid as an internal standard. The heptadecanoic acid was also used as an external standard at different concentrations to generate a standard curve for quantification. The extracted products were silylated and subjected to GC analysis (Shimadzu GC 2014) using a Restek XTI-5 fused silica capillary column (30 m x 0.25 mm, film thickness 0.25 μm) under the same conditions as for GC-MS except that the detecting port was set at 240°C and flame ionization detector (FID) was used. The 10-HSA peak area was quantified by comparing to the internal standard and the standard curve.

### 2.7. Flavin adenine dinucleotide (FAD) assay

We followed the protocol of Joo *et al* [35] to see if the NcOhy1 or NcOhy2 recombinant protein binds FAD. One mg of purified NcOhy1 or NcOhy2 in a 1.5 mL Eppendorf tube was heated at 100°C for 15 min. The denatured protein was removed by centrifugation at 13,000 ×g for 10 min followed by scanning of the supernatant from 300–600 nm using UV-1601 spectrophotometer (Shimadzu). A positive control was included where FAD solution was added to the purified enzyme prior to heating the sample to aid in locating the maximum absorption peaks at 374 and 446 nm.

### 2.8. 2^o^-ADH activity assay

The activity of Nc2^o^-ADH was monitored spectrophotometrically at 340 nm based on the reduction of cofactor NAD^+^ to NADH [70]. The assay was done at room temperature in a mixture consisting of 100 mM sodium borate buffer, pH 9.5, 0.1% Brij 56, 6 mM NAD^+^, 300 μM 10-HSA, and 5 μg purified Nc2^o^-ADH protein. Absorbance at 340 nm was recorded for 1 min and the reaction rate was calculated from the slope of the trace showing the increase in absorbance over time. Specific activity was calculated from the amount of NADH produced per min per mg enzyme.

The effects of pH on Nc2^o^-ADH activity in catalyzing oxidation of 10-HSA to 10-KSA as well as reduction of 10-KSA to 10-HSA were examined. The ability of Nc2^o^-ADH to oxidize 10-HSA to 10-KSA was carried out in the presence of 20 μg purified Nc2^o^-ADH, 2.5 mM NAD^+^, 300 μM 10-HSA, 0.1% Brij 56, and a selected buffer in a reaction volume of 400 μL. The buffers used were 100 mM of glycine-HCl, pH 2.5; 100 mM sodium acetate, pH 4.0; 100 mM MES pH 6.0; 100 mM HEPES, pH 7.8; 100 mM sodium borate, pH 9.5; or 100 mM CAPS, pH 10.5. The increase in absorbance at 340 nm was monitored and the reaction rate was calculated from the slope of the trendline.

The ability of Nc2^o^-ADH to reduce 10-KSA to 10-HSA was also carried out at pH 2.5, 4.0, 6.0, 7.8, 9.5, and 10.5 under similar conditions except that NADH was used instead of NAD^+^. The decrease in absorbance at 340 nm was monitored. The reaction rate was calculated from the slope of the trendline.

### 2.9. Design of sgRNAs that specifically target coding sequence of the *Nc2*^*o*^*-ADH* gene

The sgRNA is composed of a 20-nucleotide “targeting” or “protospacer” sequence (also known as crRNA that is homologous to a region in the *2*^*o*^*-ADH* target) and a scaffold sequence needed for Cas9 binding [71]. The scaffold is derived from endogenous bacterial tracrRNA, which is a part of the CRISPR/Cas9 expression plasmid used in this experiment. Potential protospacer sequences were created by taking the 20 nucleotides found upstream of a protospacer-adjacent motif (PAM) with NGG sequence in the *2*^*o*^*-ADH* gene. Each potential protospacer sequence was evaluated for the relative off-target possibilities by running a nucleotide BLAST against the first draft genome of *N*. *cholesterolicum* NRRL5767. The potential protospacers were ranked by predicted specificity and suggested likely off-target sites. An additional strategy for minimizing off-target effects was having a G in position 1 and A or T at position 17 of the protospacer, which are known to exhibit better specificity [72]. Three optimal protospacer sequences were selected as potential guide RNAs. Only the protospacer sequence located near 5’ end of the *Nc2*^*o*^*-ADH* gene is reported in this study. A pair of 24-nucleotide long oligos (with complementary sequence at the 20-nucleotide protospacer region) were custom synthesized by Integrated DNA Technologies, Inc.: 5’ACGCGCCTATCAGACCGCCTATCA3’ and 5’AAACTGATAGGCGGTCTGATAGGC3’. Each of the oligo has a 4-nucleotide sticky ends with ACGC on the forward oligo and AAAC on the reverse oligo to facilitate the insertion into a CRISPR vector after digestion with BbsI, a Type IIS restriction enzyme.

### 2.10. Creation of pCRISPomyces-2/sgRNA chimeric plasmid by Golden Gate Assembly

The expression vector pCRISPomyces-2 was selected as the CRISPR/Cas9 vector for *in vivo* expression. We followed the Golden Gate Assembly protocol provided by Addgene (https://media.addgene.org/data/plasmids/61/61736/61736-attachment_W-6Pygrpn0iT.pdf) to create pCRISPomyces-2/sgRNA chimeric plasmid construct. Prior to Golden Gate Assembly, the protospacer sequence that specifically targets the 5’ nucleotide coding sequence of the *2*^*o*^*-ADH* gene was generated by annealing the pair of the 24-nucleotide oligos, which are offset by 4-nucleotides, to generate sticky ends. The annealing mixture consisted of 5 μM of each oligo and 30 mM HEPES, pH 7.8 in a volume of 100 μL. The solution was heated to 95°C then ramped down to 4°C over 20 min. Golden Gate Assembly reaction consisted of 0.7 μM of pCRISPomyces-2, ~2.1 μM of protospacer insert, 10 units of BbsI, 400 units of T4 DNA ligase, and 1x T4 ligase buffer in a volume of 20 μL. The tube was placed in a thermal cycler (Mastercycler, eppendorf) at 37°C for 10 min then at 16°C for 10 min to constitute one cycle; this process was repeated for 10 cycles. Next, the temperature was raised to 50°C for 5 min (this favors BbsI digestion of pCRISPomyces-2; the pCRISPomyces-2/sgRNA no longer contains BbsI site) and then 65°C for 20 min to inactivate BbsI. Only pCRISPomyces-2/sgRNA chimeric plasmids with correct protospacer insertion would remain intact as double-stranded circular plasmids and ready for transformation [59].

### 2.11. Transformation of the Golden Gate Assembly product into *E*. *coli* NEB 5-α competent cells and screening of transformants

We followed the manufacturer’s protocol (New England BioLabs) to carry out transformation by heat shock. The Golden Gate Assembly reaction (~15 ng chimeric plasmid) was mixed with 70 μL of *E*. *coli* NEB5-α competent cells and placed in ice for 30 min followed by heat shock at 42°C for 30 sec. The tube was then placed back in ice for 5 min. Next, 950 μL of SOC medium was added to the tube and placed at 37°C for an hour. Transformants were selected by plating onto LB agar plates containing 50 μg/mL apramycin and pre-coated with 10 μL of 0.5 M IPTG and 120 μL of 20 mg/mL X-gal. The plates were incubated at 37°C overnight.

Chimeric plasmids were isolated from a few randomly selected white colonies by alkaline lysis [67] and further purified using QIAprep 2.0 Spin Miniprep Column. The DNA samples were sent to Laragen for DNA sequencing of both strands to confirm proper insertion of the protospacer. Sequencing primers (forward primer ACGGCTGCCAGATAAGGCTT and reverse primer TTCGCCACCTCTGACTTGAG) were located up and downstream from the two original BbsI sites in pCRISPomyces-2 [59].

### 2.12. Transformation of the pCRISPomyces-2/sgRNA construct into *N*. *cholesterolicum* NRRL5767 by electroporation

The correct construct confirmed by DNA sequencing was transformed into *N*. *cholesterolicum* NRRL 5767 by electroporation. We adapted Dhakal *et al*’s protocol [73] to prepare electrocompetent cells with minor changes to optimize the electroporation efficiency. One mL overnight culture of *N*. *cholesterolicum* NRRL5767 was inoculated into 100 mL of BHI medium containing 1% glycine/0.25% tween 80. The culture was incubated at 30°C and 200 rpm until OD_600_ reached ~1.2. Next, the cells were harvested by centrifugation at 2,400 xg 15°C for 10 min. The cell pellet was suspended in deionized water at room temperature and centrifuged again, this process was repeated twice. The cell pellet was then suspended in 10% glycerol at room temperature and centrifuged. Finally, the cell pellet was suspended in 0.75 mL10% glycerol and transferred to an Eppendorf tube. The tube was placed on ice and the competent cells were used immediately for electroporation. The electrocompetent cells (40 μL) were gently mixed with purified pCRISPomyces-2/sgRNA (~5.7 μg) and placed into a 1-mm electroporation cuvette (Bio-Rad). Electroporation was carried out at 25 μF, 100 Ω, and 1.25 kV for ~5 msec [73] using Gene Pulser II (Bio-Rad). In a control tube, electrocompetent cells were gently mixed with purified pCRISPomyces-2 (~4.5 μg). In another control group, electrocompetent cells were gently mixed with deionized water.

Following electroporation, the cells were placed in 400 μL BHI medium and incubated at 30°C for 2 hrs. Cells were then plated onto BHI/agar plates containing apramycin (50 μg/mL) to select transformants. Untransformed cells were used as a control and were also placed on apramycin selection plates to ensure that there was no growth on the plates. The plates were incubated at 30°C for 3–5 days. The transformants were subjected to colony PCR to check for the presence of the protospacer insert using the primer pair as described in section 2.11.

### 2.13. Screening of 2^o^-ADH knockout mutants by bioconversion reaction

The transformants that harbor the pCRISPomyces-2/sgRNA construct were tested for the lack of 2^o^-ADH activity. Each transformant was grown in 5 mL LB medium containing 50 μg/mL of apramycin at 30°C overnight. The following day, 10 mL LB was added to the culture and incubation continued for about 10 hours. The culture was harvested and resuspended in 1 mL of 100 mM sodium phosphate pH 6.8 [12] and transferred into a 1 dram glass vial. Five μL of OA was either added or omitted and capped. The mixture was incubated at 37°C, 250 rpm for 6–8 hours. Wild-type *N*. *cholesterolicum* NRRL5767 or wild-type *N*. *cholesterolicum* NRRL5767 transformed with parent pCRISPomyces-2 (clone 1-p-11) was included as a control (OA was either added or omitted). The bioconversion products were then extracted with ethyl ether and analyzed by TLC as described in section 2.6.1.

## 3. Results and discussions

### 3.1. Identification of putative *oleate hydratase* and *2*^*o*^*-ADH* genes in the genome of *N*. *cholesterolicum* NRRL5767

Two annotated *Ohase* genes located at different loci, PROKKA_00231(*NcOhy1*, GenBank: KU953947, https://www.ncbi.nlm.nih.gov/nuccore/1187633312) and PROKKA_02988 (*NcOhy2*, GenBank: KU953948, https://www.ncbi.nlm.nih.gov/nuccore/KU953948), were identified in the genome of *N*. *cholesterolicum* NRRL5767 by a homology search using the amino acid sequence of Ohase (OhyA) of *Elizabethkingia meningoseptica* (GenBank: GQ144652.1). The NcOhy1 shares 42.61% amino acid identity with OhyA; NcOhy2 shares 32.4% with OhyA; and NcOhy1 shares 33.33% with NcOhy2 (See S1 Fig for protein sequence alignment). These two genes were cloned and functional recombinant proteins were shown to possess Ohase activity (See section 3.2).

Similarly, an annotated putative *3-hydroxybutyryl-CoA dehydrogenase* gene located at PROKKA_03439 of *N*. *cholesterolicum* NRRL5767 (*Nc2*^*o*^*-ADH*, GenBank: KU953949.1, https://www.ncbi.nlm.nih.gov/nuccore/1187633316) was identified through a homology search with the amino acid sequence of 2^o^-ADH of *M*. *luteus* WIUJH20 [27] (Accession E2D104). The Nc2^o^-ADH shares 47.37% amino acid identity with Ml2^o^-ADH (See S2 Fig for protein sequence alignment). This gene was cloned and the functional recombinant protein possessed 2^o^-ADH activity (See section 3.3).

### 3.2. Characterization of recombinant Ohases from *N*. *cholesterolicum* NRRL 5767

**Fig 1:**
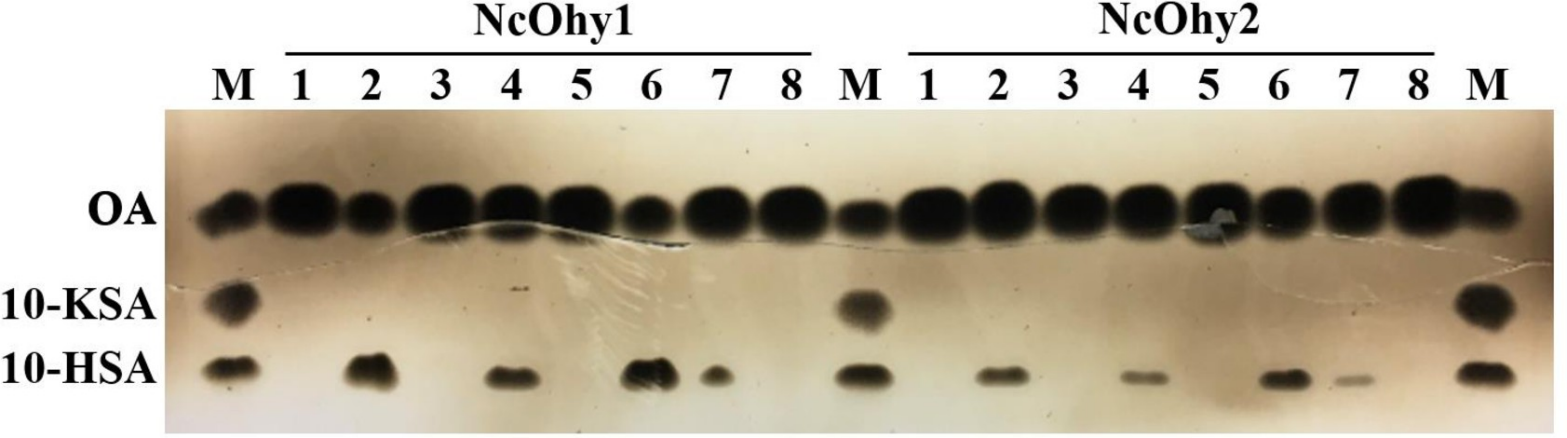
TLC analysis of ohase activity of NcOhy1 and NcOhy2. Four mg of each isozyme was used per reaction. Lanes M are fatty acid standard. Lanes 1–8 under NcOhy1 and NcOhy2 are the corresponding lanes with the same substrates but different isozymes, respectively. Lanes 1 and 2 are reactions with OA in the absence or presence of NcOhy1 or NcOhy2. Lanes 3 and 4 are reactions with LA in the absence or presence of NcOhy1 or NcOhy2 (LA and OA have similar R_f_ values; 10OH-12OD and 10-HSA have similar R_f_ values). Lanes 5–8 are reactions with OA in the presence of each enzyme at pH 4.0, 6.5, 8.0, and 9.5, respectively.

The cloned *NcOhy1* gene is 1767 bp long and the deduced protein consists of 588 amino acid residues with a predicted molecular mass of 67 kDa and pI of 5.32 [31]. The *NcOhy2* gene is 1680 bp long and its deduced protein consists of 559 amino acid residues with a predicted molecular mass of 63.7 kDa and pI of 5.25 [74]. Both recombinant proteins were shown to possess Ohase activity as determined by their ability to convert OA to 10-HSA and LA to 10OH-12OD (Fig 1). Both NcOhy1 and NcOhy2 are capable of transforming OA to 10-HSA (lane 2) and LA to 10OH-12OD (lane 4) demonstrating that both recombinant proteins are active. The NcOhy1 is more active than NcOhy2 as more 10-HSA and10OH-12OD were produced, which agrees with the specific activity data shown in Table 2. Additionally, both enzymes are more active toward OA substrate than LA, which agrees with the specific activity data shown in Table 2 and the finding of Koritala *et al* [12, 13].

**Table 2:** Substrate, bioconversion product, and specific activity of Ohase isozymes (NcOhy1 and NcOhy2) and Nc2^o^-ADH.

Substrate	Product	*Specific activity (nmol/min/mg)
NcOhy1		
Oleic acid	10-hydoxystearic acid	2410 ± 45.5
Linoleic acid	10-hydroxy-12(Z)-octadecenoic acid	601 ± 50.1
NcOhy2		
Oleic acid	10-hydoxystearic acid	610 ± 35.2
Linoleic acid	10-hydroxy-12(Z)-octadecenoic acid	133 ± 10.4
Nc2^o^-ADH		
10-hydroxystearic acid	10-ketostearic acid	5 x 10^5^ ± 150
Ricinoleic acid	12-keto-9(Z)-octadecenoic acid	2.2 x 10^4^ ± 210

* Average of triplicates

The pH dependence of NcOhy1 and NcOhy2 were performed under various pH using OA as the substrate (Fig 1, lanes 5–8). The NcOhy1 and NcOhy2 are most active at pH 6.5 as most 10-HSA product was detected at this pH (lane 6). At pH 4 (lane 5) and pH 9.5 (lane 8), no activity was detected as 10-HSA band was not detected. These results agree with other reported Ohases [19].

The putative 10-HSA and 10OH-12OD bands from both samples were purified from the TLC plate, subjected to silylation, and structures confirmed by GC-MS (S3 Fig and S4 Fig). The discovery of two functional Ohase isozymes in *N*. *cholesterolicum* NRRL5767 may account for the high bioconversion yields of 10-HSA and 10OH-12OD from OA and LA, respectively [12, 13].

The specific activities of each enzyme toward OA or LA substrate are shown in Table 2. It is apparent that NcOhy1 is more active than NcOhy2 as the specific activity is about 4 times greater. This result is consistent with the TLC results shown in Fig 1.

Amino acid sequences of the NcOhy1 and NcOhy2 were aligned with six well-characterized Ohases using Clustal Omega multiple sequence alignment tool (alignment figure not shown). The NcOhy1 shares 42.61% amino acid identity with OhyA [44], 42.65% with OhySt [46], 42.96% with smOhyA2 [50], 51.71% with LAH [43], 76.87% with smOhyA1 [50], 33.33% with NcOhy2, and 33.33% with OhyRe [45]. NcOhy2 is nearly identical to OhyRe from *Rhodococcus erythropolis* with 99.11% homology. A comparison of consensus sequences among these Ohases is shown in Table 3. The NcOhy1’s consensus sequences are more highly conserved with OhyA, smOhyA2, LAH, and OhySt than with NcOhy2 and OhyRe. The NcOhy1 has higher Ohase activity than NcOhy2 (~4 times higher) (Table 2) which coincides with the fact that NcOhy1’s consensus sequences are more highly conserved (Table 3). A search of Ohases from *Nocardia* in Protein knowledgebase (https://www.uniprot.org/) retrieved 18 sequences (including NcOhy1 and NcOhy2). Amino acid sequence alignment revealed that 16 of the Ohases share 70–80% homology with NcOhy1. As can be seen from sequence alignment of the first 120 amino acid residues (S5 Fig), the FAD binding pocket (R_78_GGREM_83_) is totally conserved and the Rossmann fold (GSGLASX _(21)_ E) is highly conserved among these Ohases. Both NcOhy1 and NcOhy2 do not require FAD for enzyme activity even though they contain putative consensus FAD-binding pocket (NcOhy2’s putative FAD-binding pocket is less conserved). Addition of FAD in the dialysis buffer of purified NcOhy1/NcOhy2 or adding FAD cofactor in the activity assays showed no effect on NcOhy1 or NcOhy2 activity (data not shown). We did not detect FAD absorption peaks for either of the purified NcOhy1 and NcOhy2; unlike some Ohases which can be visualized by a slightly yellowish appearance or detected at 446–450 nm [33, 35].

**Table 3:** Comparison of consensus sequences among oleate hydratases.

Ohases	OhyA	SmOhyA2	LAH	SmOhyA1	NcOhy1	OhySt	OhyRe	NcOhy2
Rossmann-fold*	GSGIAGX _(21)_E	GTGLAGX _(21)_E	GSGLAGX _(21)_E	GSGLASX _(21)_E	GSGLASX _(21)_E	GSGIAGX _(21)_D	GAGIGNX _(21)_G	GAGIGNX _(21)_G
FAD-binding pocket	R_118_GGREM_123_	R_114_GGREM_119_	R_78_GGREM_83_	R_78_GGREM_83_	R_78_GGREM_83_	R_60_GGREM_65_	R_73_GGR${\mathrm{ML}}_{78}$	R_73_GGR${\mathrm{ML}}_{78}$
**Double bond of oleic acid binding site**	**E**_**122**_	**E**_**118**_	**E**_**82**_	**E**_**82**_	**E**_**82**_	**E**_**64**_	$M_{77}$	$M_{77}$
	**Y**_**241**_	**Y**_**237**_	**Y**_**200**_	**Y**_**201**_	**Y**_**201**_	**Y**_**183**_	**Y**_**205**_	**Y**_**205**_
	**Y**_**456**_	**Y**_**452**_	**Y**_**411**_	**Y**_**411**_	**Y**_**411**_	**Y**_**398**_	**Y**_**413**_	**Y**_**413**_
**Carboxylate of oleic acid binding site**	**Q**_**265**_	**Q**_**261**_	**Q**_**224**_	**Q**_**225**_	**Q**_**225**_	**Q**_**207**_	**Q**_**229**_	**Q**_**229**_
	**T**_**436**_	**T**_**432**_	**T**_**391**_	**T**_**391**_	**T**_**391**_	**T**_**378**_	$V_{393}$	$V_{393}$
	**N**_**438**_	**N**_**434**_	$H_{393}$	**N**_**393**_	**N**_**393**_	**N**_**380**_	$H_{395}$	$H_{395}$
	**H**_**442**_	**H**_**438**_	**H**_**397**_	**H**_**397**_	**H**_**397**_	**H**_**384**_	**H**_**399**_	**H**_**399**_
**References**	**Engleder *et al* [44]**	**Kang *et al* [50]**	**Volkov *et al* [43]**	**Kang *et al* [50]**	**This study**	**Park *et al* [46]**	**Lorenzen *et al* [45]**	**This study**

*Consensus sequence of Rossmann fold: GXGXXGX_**(17/23)**_E

OhyA is from *Elizabethkingia meningoseptica*

SmOhyA1 and SmOhyA2 are from *Stenotrophomonas maltophilia*

LAH is from *Lactobacillus acidophilus*

OhySt is from *Stenotrophomonas sp*. *KCTC 12332*

OhyRe is from *Rhodococcus erythropolis*

### 3.3. Characterization of 2^o^-ADH from *N*. *cholestrolecium* NRRL 5767

The *Nc2*^*o*^*-ADH* gene is 861 bp long and the deduced protein consists of 286 amino acid residues with a predicted molecular mass of ~31.2 kDa and pI of 4.95. The 2^o^-ADH from *M*. *luteus* WIUJH20 has a predicted molecular mass of ~33.7 kDa and pI of ~4.67 [27]. As stated before, they share 47.37% amino acid identity (S2 Fig).

**Fig 2:**
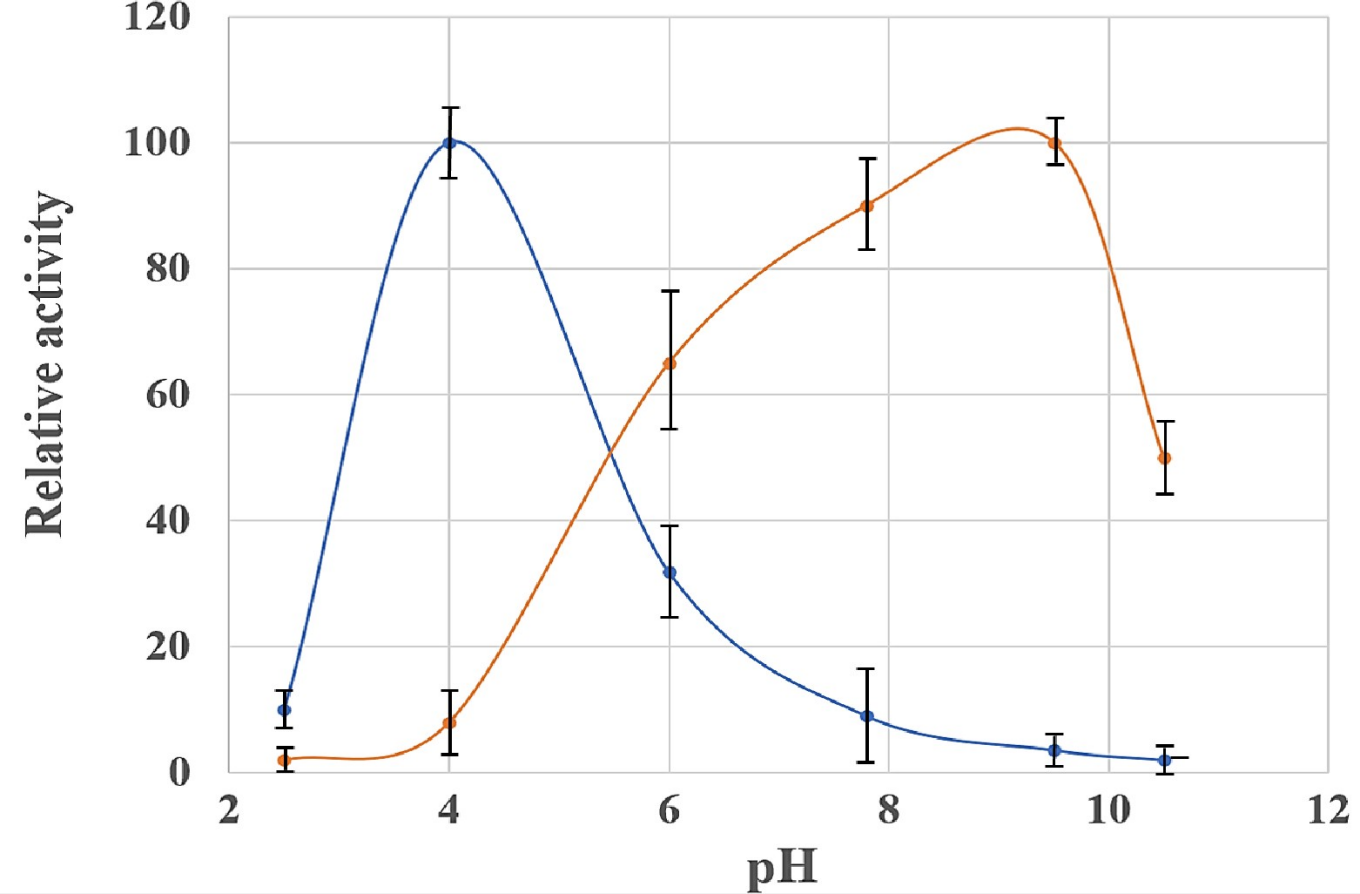
pH-dependence of recombinant Nc2^o^-ADH activities. The ability of Nc2^o^-ADH to oxidize 10-HSA to 10-KSA was carried out at pH 2.5, 4.0, 6.0, 7.8, 9.5, and 10.5 in the presence of NAD^+^ (orange). The condition that yielded the highest activity was set at 100%. The ability of Nc2^o^-ADH to reduce 10-KSA to 10-HSA was carried out at the same pH conditions in the presence of NADH (blue). These results are from triplicates.

The recombinant Nc2^o^-ADH is able to oxidize 10-HSA and ricinoleic acid to their corresponding keto derivatives as shown in Table 2. The optimal pH of the Nc2^o^-ADH enzyme to oxidize 10-HSA to 10-KSA is about pH 9.5 (Fig 2 orange) and the optimal pH of the enzyme to reduce 10-KSA to 10-HSA is at pH 4.0 (Fig 2 blue). These results agree with general trend of ADHs [51, 75].

### 3.4. *2*^*o*^*-ADH* gene knockout in *N*. *cholesterolicum* NRRL5767 by CRISPR/Cas9 genome editing technology

#### 3.4.1. Creation of a CRISPomyces-2/sgRNA chimeric plasmid that targets the *Nc2*^*o*^*-ADH* gene

Very few prokaryotic CRISPR/Cas9 vectors were commercially available. We chose the expression vector, pCRISPomyces-2 [59], because it worked in *Streptomyces* species and it is available through Addgene. *Streptomyces* and *Nocardia* are closely related in the same phylum *Actinobacteria* [76]. It is likely that the pCRISPomyces-2 vector would work in *N*. *cholesterolicum*, NRRL5767.

pCRISPomyces-2 contains several key components which are important for genome editing: (1) It incorporates a *lacZα* gene cassette flanked by BbsI sites in opposing directions to facilitate the construction of a fusion gene (for transcribing a sgRNA in host cells) by one-step Golden Gate Assembly. (2) It allows selection of colonies bearing chimeric plasmid by blue-white screening. Colonies transformed with the parent plasmid, pCRISPomyces-2, would express the Lac operon induced by IPTG and detected by X-gal as blue colonies. A successfully assembled recombinant plasmid would have no LacZ expression (as the *LacZα* would have been replaced by the protospacer sequence); the colony bearing such chimeric plasmid would appear white when treated with IPTG and X-gal. (3) It contains a *Cas9* gene under the control of rpsL (30S ribosomal protein S12) promoter. In addition, glyceraldehyde 3-phosphate dehydrogenase (GAPDH) promoter controls the expression of the sgRNA. Both of which are common in bacteria, hence Cas9 and sgRNA are likely to be expressed in *N*. *cholesterolicum* NRRL5767. (4) It contains the apramycin resistant gene to facilitate screening of transformants. (5) It contains a region expressing a temperature sensitive replication protein (pSG5) allows clearance of the pCRISPomyces-2/sgRNA following genome editing [59, 77]. This may increase CRISPR specificity by reducing the amount of time that Cas9 is available for off-target cleavage [71]. The potential protospacer that specifically targeted a region near the 5’ coding sequence of the *2*^*o*^*-ADH* gene was successfully inserted into pCRISPomyces-2 by Golder Gate Assembly as described below.

#### 3.4.2. Transformation of the Golden Gate Assembly reaction mixture into NEB 5-α competent cells and selection of transformants bearing the desired pCRISPomyces-2/sgRNA

The reaction mixture was transformed into NEB 5-α competent cells and the desired transformants were selected by blue/white screening and apramycin resistance. A total of 130 colonies were obtained with only one blue colony suggesting that the Golden Gate Assembly was efficient. Subsequently, chimeric plasmids (pCRISPomyces-2/sgRNA) were isolated from 3 white colonies (Clones #3, #40, and #129) and DNA sequencing was determined on both strands using primers flanking the protospacer sequence. The forward primer was approximately 92 bp upstream of the 5’ BbsI cloning site and the reverse primer was about 238 bp downstream of the 3’ BbsI site. All the three chimeric plasmids were assembled correctly as determined by DNA sequencing (data not shown) demonstrating the successful construction. The chimeric plasmid from clone #3 was selected for electroporation.

#### 3.4.3. Transformation of the pCRISPomyces-2/sgRNA construct into *N*. *cholesterolicum* NRRL5767 by electroporation

**Fig 3:**
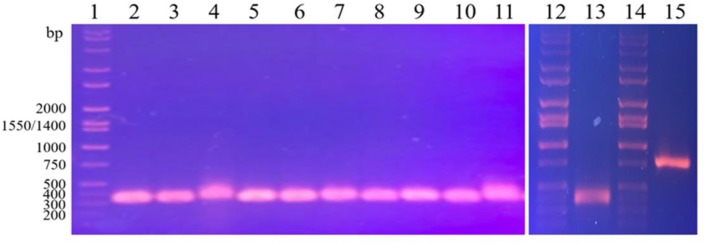
Screening of *N*. *cholesterolicum* NRRL5767 transformants by colony PCR. Lanes 1, 12, and 14: Hi-Lo DNA marker; lanes 2–11: selected apramycin resistant *N*. *cholesterolicum* NRRL5767 transformants were the sources of DNA template; lane 13: purified pCRISPomyces-2/sgRNA was used as DNA template; lane 15: purified pCRISPomyces-2 was used as DNA template.

We adapted the protocol of Dhakal *et al* [73] to prepare competent cells for electroporation of *N*. *cholesterolicum* NRRL5767. Several conditions were tested. In the first three trials, the transformation efficiencies were poor with only a handful of transformants. It is likely that the low efficiency was due to significant cell clumping at each step. In Dhakal *et al*’s protocol, all steps were carried out on ice and all solutions were kept ice-cold. We noticed that the cells were less clumpy at room temperature and yielded better transformation efficiency. More than 160 transformants were obtained from the last two trials by carrying out washing steps at room temperature. Each of the transformants was tested for the presence of the protospacer insert by colony PCR using primers flanking the protospacer insertion site. The clones containing the desired sgRNA (pCRISPomyces-2/sgRNA) should produce a PCR fragment with an expected size of 353 bp long. The clones containing the parent pCRISPomyces-2 should produce a band of 790 bp long. Fig 3 shows a sample result of colony PCR analyzed by agarose gel electrophoresis. A ~400 bp fragment was detected in Fig 3 lanes 2–11 suggesting that these transformants bear pCRISPomyces-2/sgRNA. Purified pCRISPomyces-2/sgRNA (lane 13) and pCRISPomyces-2 (lane 15) were used as control templates in PCR. As expected, a ~400 bp and a ~800 bp fragment were produced, respectively. Some of the transformants although resistant to apramycin did not yield any PCR products (results not shown). It should be noted that the wild-type *N*. *cholesterolicum* NRRL5767 is sensitive to apramycin. Colony PCR method is inherently tricky, it is possible that some apramycin resistant transformants gave false negatives in colony PCR. Transformants that were positive in colony PCR were screened for knockout mutations by their ability to convert OA to 10-HSA. Later, we decided to screen the remainder of the apramycin resistant transformants even though they were negative by colony PCR.

#### 3.4.4. Screening of *2*^*o*^*-ADH* knockout mutants by biotransformation

**Fig 4:**
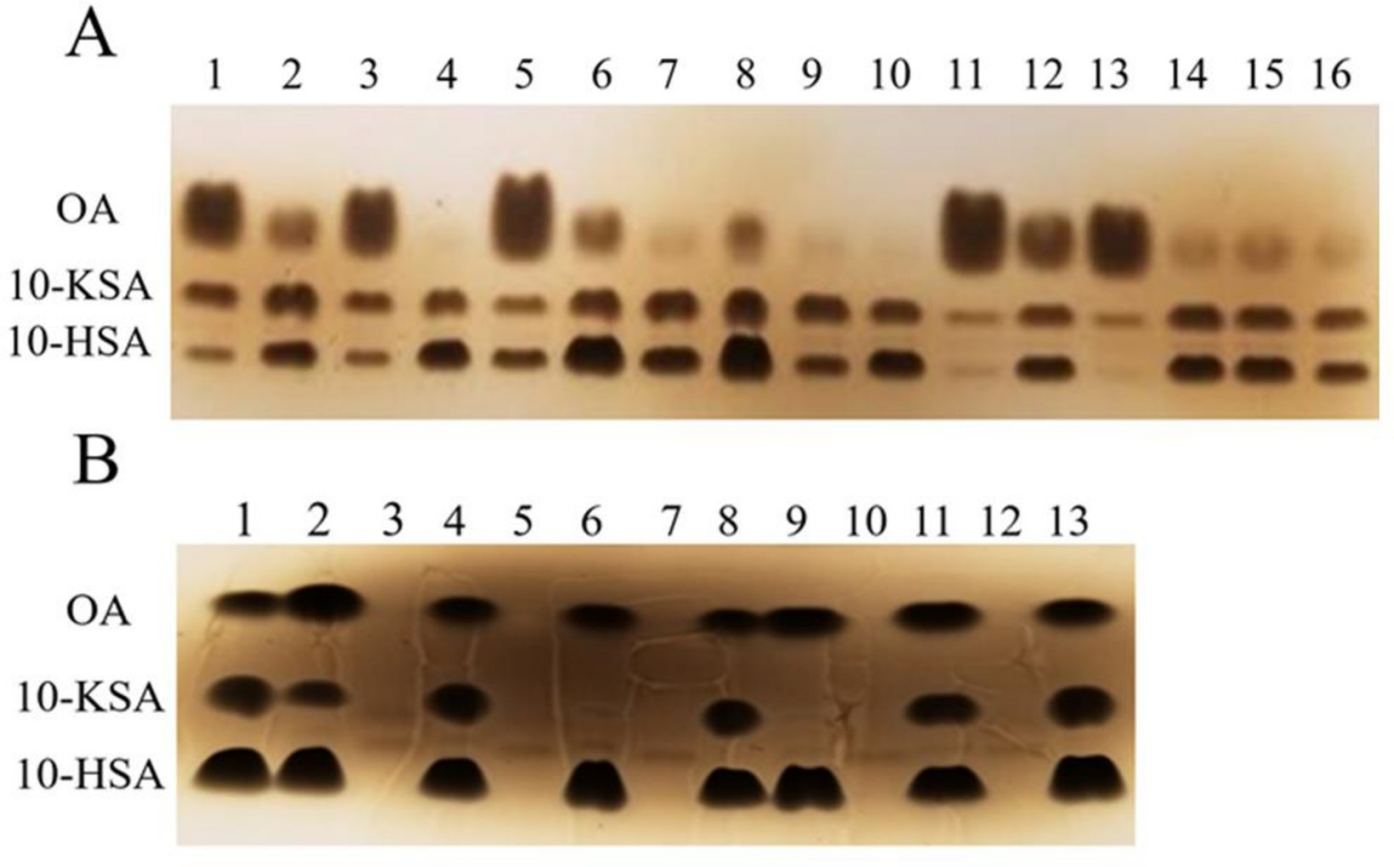
TLC analysis of bioconversion products of *N*. *cholesterolicum* NRRL 5767 transformants. (A) Lane 8 contains fatty acid standard. Lanes 1–7 and 9–16 are individual transformants with OA as substrate. (B) Lanes 1, 8, and 13 contain fatty acid standard. Lanes 2 and 3 are *N*. *cholesterolicum* NRRL5767 with and without OA, respectively. Lanes 4 and 5 are transformant 1-3-3 with and without OA, respectively. Lanes 6 and 7 are knockout mutant 1-3-17 with and without OA, respectively. Lanes 9 and 10 are knockout mutant 2-3-52 with and without OA, respectively. Lanes 11 and 12 are clone 1-p-11 with and without OA, respectively.

The transformants that bear pCRISPomyces-2/sgRNA were tested for the lack of 2^o^-ADH activity. The wild-type *N*. *cholesterolicum* NRRL5767 and/or the transformant that bears parent pCRISPomyces-2 (clone 1-p-11) were included as positive controls. The positive control cultures should be able to convert OA to produce both 10-HSA and 10-KSA whereas the knockout mutants should convert OA to a single product, 10-HSA. More than 160 transformants were screened, most of them produced both 10-HSA and 10-KSA as observed on TLC plates. Fig 4A is a representative of such screening. All the clones shown in Fig 4A produced varying degree of 10-HSA and 10-KSA suggesting that both Ohase and 2^o^-ADH are active. Among all the clones screened, two knockout mutants were discovered (clones 1-3-17 and 2-3-52). As can be seen in Fig 4B, both mutants 1-3-17 (lane 6) and 2-3-52 (lane 9) converted OA to 10-HSA solely; 10-KSA was undetectable in these lanes. These results are reproducible and clearly demonstrated that they lack the 2^o^-ADH activity. In the absence of OA substrate (lanes 7 and 10), the OA, 10-KSA, and 10-HSA bands were not detected. Both positive control clones, wild-type *N*. *cholesterolicum* NRRL5767 (lane 2) and clone 1-p-11 (lane 11), produced both 10-HSA and 10-KSA. Taken together, these results demonstrated that the *2*^*o*^*-ADH* gene knockout is successful in clones 1-3-17 and 2-3-52.

### 3.5. Validation of *2*^*o*^*-ADH* gene knockout in *N*. *cholesterolicum* NRRL5767 mutants

At the protein level, we have demonstrated that mutants 1-3-17 and 2-3-52 lack 2^o^-ADH enzyme activity through bioconversion screening (Fig 4B). To see if an on-target mutation took place on the *2*^*o*^*-ADH* gene, genomic DNA was isolated from the knockout mutants, wild-type *N*. *cholesterolicum* NRRL5767, and clone 1-p-11. The integrity of these genomic DNA is shown in S6 Fig. Very little smear was seen although a small amount of RNA was at the bottom of the gel. Our plan was to amplify a DNA fragment that covers the mutation and determine its sequence.

**Fig 5:**
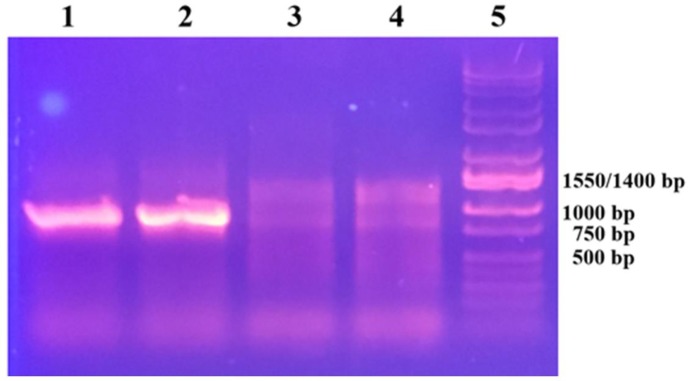
Agarose gel electrophoresis of PCR products using primer pair 1 and 13. Lane 1: genomic DNA from *N*. *cholesterolicum* NRRL5767 was the template. Lane 2: genomic DNA from clone 1-p-11 was the template. Lane 3: genomic DNA from the knockout mutant 1-3-17 was the template. Lane 4: genomic DNA from the knockout mutant 2-3-52 was the template. Lane 5: Hi-Lo DNA marker.

To begin, PCR was performed using the forward primer (primer 1) and reverse primer (primer 13) to see if we were able to amplify the entire coding region of the *Nc2*^*o*^-*ADH* (861 bp) from the genomic DNA of the two knockout mutants, the wild-type *N*. *cholesterolicum* NRRL5767, and clone 1-p-11. The results are shown in Fig 5. As expected, a band located between 750 and 1,000 bp was amplified from the wild-type genomic DNA and clone 1-p-11 (Fig 5, lanes 1 and 2) but there was no amplification of the *Nc2*^*o*^*-ADH* gene from the genomic DNA of either of the knockout mutants (Fig 5, lanes 3 and 4). These results suggested that the 5’coding sequence, at least the part covered by the primer 1 (nucleotide position 1–25) has been mutated/deleted. It was a surprise because the error-prone NHEJ typically results in small insertions and/or deletions (indels) at the site of the break [56] and the protospacer sequence target site is at nucleotide position 58–77. This suggests the deletion/mutation occurred at least 50 bp beyond the 5’ upstream of the protospacer sequence site.

To determine how far downstream or upstream from the protospacer sequence the *Nc2*^*o*^*-ADH* gene has been deleted/mutated, additional primers located throughout the coding region and 5’ upstream were synthesized for use in PCR (Table 4).

**Table 4:** Nucleotide sequence of primers and their relative locations within the coding region and its 5’ upstream of the *Nc2*^*o*^-*ADH* gene.

Primers number and direction	Nucleotide sequence and its position
Within the *Nc2*^*o*^*-ADH* gene	
1 (5’ forward)	^1^ATGACTGAACTGAAGCAGATCACCG^25^
2 (forward) (protospacer sequence)	^58^GCCTATCAGACCGCCTATCA^77^
3 (forward)	^81^ATTCGACGTCGTCGCGTA^98^
4 (forward)	^106^AACGCCGAGGTCATCAAGA^124^
5 (forward)	^130^AAGGCTCGGTTCGACTCGTT^149^
6 (forward)	^213^GCAACGTATTACGTACTCGTACG^235^
7 (forward)	^262^GATCTTGTCATCGAGGCAATTCC^284^
8 (forward)	^330^AGTAGCTCCTGAGCACACGGT^350^
9 (reverse)	^350^ACCGTGTGCTCAGGAGCTACT^330^
10 (forward)	^373^CTTCTGCCGAGCGACCTCAA^392^
11 (forward)	^452^TCAACAACACTGCCGAGGTCAT^473^
12 (forward)	^655^GTCGACAAGACGTGGCGTAT^674^
13 (3’ reverse)	^861^TCAGCCTTTGTAGTTGTAGAAGCCC^836^
5’ upstream to the *Nc2*^*o*^*-ADH* gene	
14 (forward)	^-90^ATCACGGCTGACGAACTCAC^-71^
15 (forward)	^-145^GCGAACGATTTCCAACGTGC^-126^
16 (forward)	^-206^GGATTGCGCAAATCTTGCTCG^-186^
17 (forward)	^-265^CGAGCCCAGCGAATGTTTCC^-246^
18 (forward)	^-445^GAGCTACTGCTCATGGTGTACAAC^-422^
19 (forward)	^-623^ATCGGACCAAGAGTTGGGTC^-604^
20 (forward)	^-786^AATCGACGCATGCGTGCGAA^-767^
21 (forward)	^-961^GAGGAATTGTCCTGGAGGCAT^-941^

*Nc2*^*o*^-*ADH* is 861 bp long. The superscript with positive numerals indicates the position in the coding sequence of *Nc2*^*o*^*-ADH*. The superscript with negative numerals indicates the position at the 5’ upstream of *Nc2*^*o*^*-ADH*.

**Fig 6:**
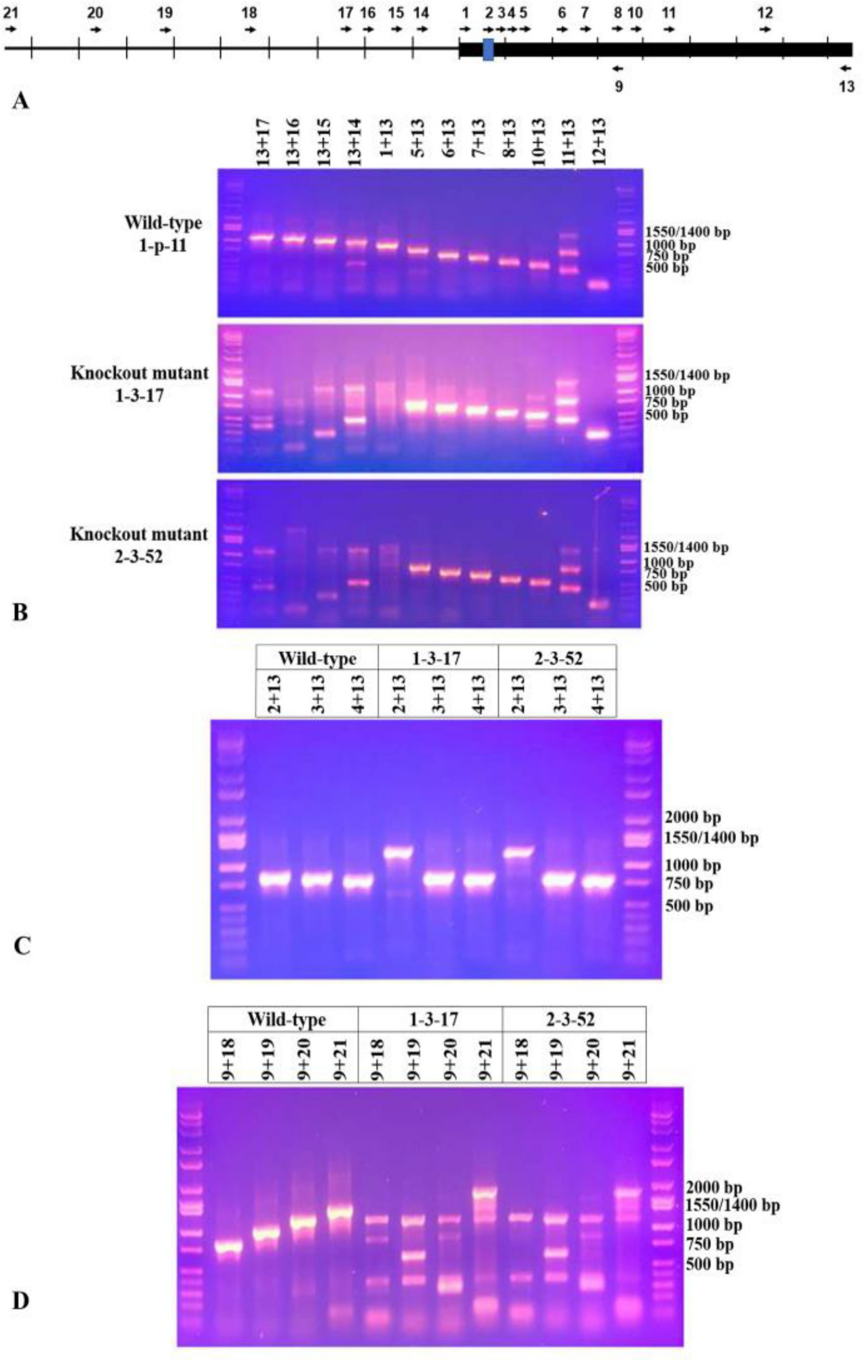
Agarose gel electrophoresis of PCR products using primers located throughout the coding region and its 5’ upstream of *Nc2*^*o*^*-ADH*. (A) shows the location and direction of the PCR primers. The thick bar is the *NC2*^*o*^*-ADH* coding region and the blue shows the protospacer site. The scale is 100 bp long. (B-D) are agarose gel images of PCR products. The templates used were genomic DNA isolated from clone 1-p-11, knockout mutants 1-3-17 and 2-3-52. Primer pairs used are indicated on top of each lane.

Fig 6A is a map showing the location and direction of the primers listed in Table 4. We expected to see DNA fragments of 1126 bp, 1076 bp, 1006 bp, 951 bp, and 861 bp when the primer pairs of 13+17, 13+16, 13+15, 13+14, and 1+13 were used to amplify genomic DNA from clone 1-p-11. As predicted, these fragments were produced (Fig 6B). However, none of these fragments were produced from the knockout mutants except for a common band of ~1.1 kb appeared in all the lanes using primer pair 13+17. The ~1.1 kb fragments amplified from the knockout mutants were later proven to be different from the wild-type (clone 1-p-11) by DNA sequencing (see S8 Fig). The results suggest that the mutation/deletion had occurred at the coding sequence near 5’ end and its 5’ upstream to the position -265. It is interesting to note that the PCR banding patterns in lanes with primer pairs 13+17 and 13+16 are different for the two knockout mutants (Fig 6B) suggesting that the 2 mutants may be different.

We expected to see PCR fragments of 731 bp, 630 bp, 599 bp, 531 bp, 488 bp, 409 bp, and 201 bp when the primer pairs of 5+13, 6+13, 7+13, 8+13, 10+13, 11+13, and 12+13 were used to amplify genomic DNA of clone 1-p-11. As predicted, these fragments were produced (Fig 6B). All the fragments were also produced in both knockout mutants suggesting that mutation/deletion didn’t occur between the nucleotide position 130 and to the 3’end of the *Nc2*^*o*^*-ADH* coding sequence. Two non-specific bands of about 750 bp and 1,300 bp were also produced in the lanes using primer pair 11+13 in all three genomic DNA samples.

Possible mutations/deletions between the protospacer sequence and position 130 (i.e., nucleotide position 58–130) were tested with additional primer pairs located in this region (2+13, 3+13, and 4+13). Fig 6C shows PCR results using these primer pairs. We expected to see PCR fragments of 804 bp, 781 bp, and 756 bp with 1-p-11 DNA template. As predicted, these fragments were produced (Fig 6C). Instead of producing an 804 bp fragment, a different size fragment of about 1300 bp was produced in both mutants with primer pair 2+13. These results suggest that the protospacer sequence site (where primer 2 located) had been mutated/deleted in both mutants (Fig 6C). The correct PCR size fragments were produced by both mutants with primer pairs 3+13 and 4+13 suggesting that the region immediately downstream of the protospacer sequence site is not mutated (Fig 6C). The results suggest that genome editing was on-target (at least on the 3’ side of the protospacer) and the break is likely to occur a few nucleotides upstream of the PAM.

**Fig 7:**
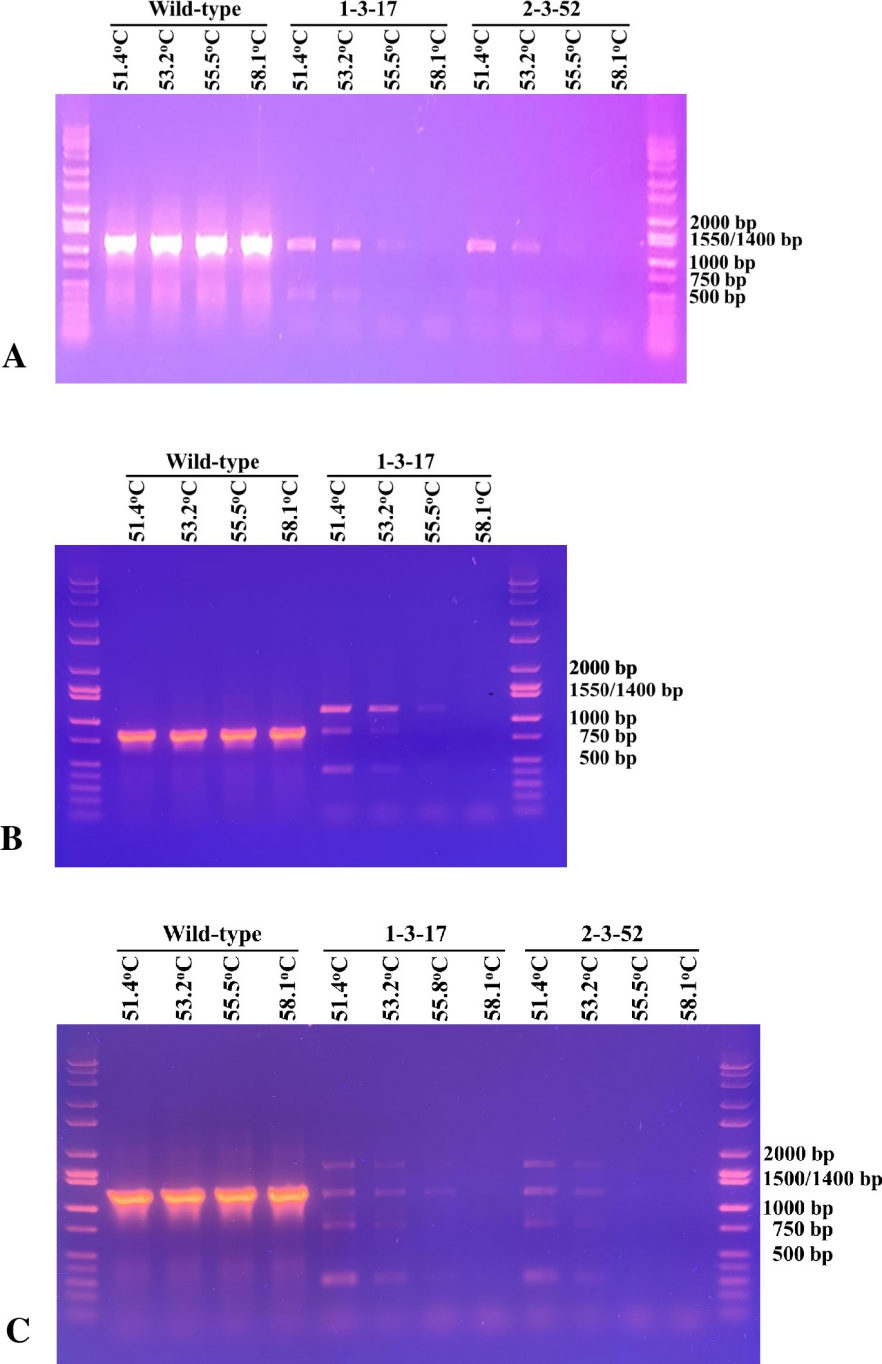
PCR amplifications under stringent annealing temperatures. (A) amplification of the 1.1 kb fragments using primer pair 13+17. (B) amplification of the 0.8 kb fragments using primer pair 9+18. (C) amplification of the 1.2 kb fragments using primer pair 9+20. The templates used were genomic DNA isolated from clone 1-p-11, knockout mutants 1-3-17 and/or 2-3-52. Annealing temperatures used are indicated on top of each lane.

From Fig 6B (lanes 2–6), we conclude that the mutation/deletion occurred at the upstream of the protospacer sequence site up to nucleotide position -265. To see if mutations occur further upstream, additional PCRs were performed using primers located further upstream (primer pairs 9+18, 9+19, 9+20, 9+21). Fig 6D shows the PCR results using these primer pairs. The expected PCR fragments with 1-p-11 genomic DNA template are 795 bp, 973 bp, 1136 bp, and 1311 bp, respectively. As predicted, these fragments were produced (Fig 6D). However, most of these fragments were not produced in the knockout mutants suggesting mutation/deletion occurred further upstream to nucleotide position -961. Nevertheless, there is a common band of ~0.8 kb appeared in the wild-type and mutant 1-3-17 using primer pair 9+18 with lower intensity in the mutant. There is another band of ~1.2 kb appeared in the wild-type and both mutants using primer pair 9+20 with lower intensity in the mutants. These fragments appeared in the mutants were later proven to be non-specific (see Fig 7B and 7C). It should be noted that the PCR banding patters using the primer pair 9+18 are different for the two knockout mutants (Fig 6D) suggesting that the 2 mutants may be different.

To investigate the difference of the ~1.1 kb DNA fragments produced by the primer pair 13+17 with each of the genomic DNA template (Fig 6B), we performed PCR with increasing annealing temperatures from 51.4°C to 58.1°C. When the 1-p-11 genomic DNA was the template, strong intensity of the ~1.1 kb fragment was produced at each temperature (Fig 7A). For the knockout mutants, the bands are visible (weaker intensity than the 1-p-11) at 51.4°C and 53.2°C but fades out at the higher annealing temperatures. The primers appear to be less specific toward the knockout mutants suggesting that the ~1.1 kb fragments were off-target amplifications. Each of the fragment was gel purified and sent to Laragen for DNA sequencing of both strands using primers 13 and 17. As expected, the nucleotide sequence of the fragment produced by the 1-p-11 DNA is nearly identical to the *Nc2*^*o*^*-ADH* gene and its upstream (S7 Fig). The fragment produced by each of the knockout mutants has nearly identical nucleotide sequences, but they do not match the *Nc2*^*o*^*-ADH* gene. A BLAST search of the sequence reveals that the sequence encodes a part of glutamate synthase large subunit. Nucleotide sequence alignment of the PCR fragments from the two mutants with homologous sequence found in *N*. *cholesterolicum* NRRL5767 genome (range 507572–506579) and *Rhodococcus erythropolis* strain X5 chromosome CP044284.1 (range 65626–66619) is shown in S8 Fig.

Similarly, we investigated the common ~0.8 kb appeared in the wild-type and mutant 1-3-17 with primer pair 9+18 (Fig 6D) and the ~1.2 kb appeared in the wild-type and both mutants (Fig 6D) by performing PCR at increasing annealing temperatures. The results are shown in Fig 7B and 7C. When the wild-type 1-p-11 genomic DNA was the template, strong intensity of the ~0.8 kb fragment was produced at each temperature with primer pair 9+18. For the 1-3-17 knockout mutant, the bands are faint at 51.4°C and 53.2°C and nearly undetectable at the higher annealing temperatures (Fig 7B). Similarly, when the 1-p-11 genomic DNA was the template, strong intensity of the ~1.2 kb fragment was produced at each temperature with primer pair 9+20. For the knockout mutants, the bands are faint at 51.4°C and 53.2°C and fades out at the higher annealing temperatures (Fig 7C). The results demonstrated that the ~0.8 kb and 1.2 kb fragments seen in the mutants were non-specific amplifications.

From the above PCR analyses and DNA sequencing data (Fig 6B–6D, Fig 7A–7C, S7 Fig and S8 Fig), it is clear that the gene knockout was successful. The large region of deletion/mutation to the 5’ of the protospacer sequence site was a surprise. On one hand, the genome editing was on-target (Fig 6C) and the cleavage by Cas 9 most likely took place a few nucleotides 5’ to the PAM as expected. On the other hand, the NHEJ did not result in small indels at the site of the break. The deleted/mutated region includes an annotated gene, PROKKA_03440 HTH-type transcriptional repressor KstR, which is adjacent to the *Nc2*^*o*^-*ADH* gene. A possible explanation of the large deletion/mutation is that in addition to the on-target mutation at the protospacer site, an off-target mutation also occurred at a 5' site upstream of the *Nc2*^*o*^*-ADH* gene. Further investigation is needed to determine the exact location of the mutation in the knockout mutant genome. In the literature, large deletions and complex rearrangements induced by CRISPR/Cas9 genome editing have been reported in other species [78–80]. Shin *et al* reported the molecular consequences on targeted sites with single sgRNAs and identified prevalent asymmetric deletions (up to 600 bp) in the mouse genome [78]. Using mouse embryonic stem cells, hematopoietic progenitors, and a differentiated human cell line in their investigation, Kosicki *et al* reported that about 20% of the CRISPR-induced DSBs resulted in significantly larger deletions (>250 nt) and more complex genomic rearrangements [79]. Some of these events were shown to extend up to several kilobases from the PAM site [79]. Thomas *et al* discussed that the larger structural variation highlighted by Kosicki *et al* may be less common and that multiple DSBs within the same chromosome associated with either on-target or combined on- and off-target activity could result in large deletions and complex rearrangements [80].

In conclusion, this is the first report of molecular cloning and expression of two functional Ohase isozymes (NcOhy1 and NcOhy2) and a functional NAD^+^-dependent 2^o^-ADH from *N*. *cholesterolicum* NRRL5767. The presence of two oleate hydratase isozymes may explain the high conversion yield of 10-HSA from OA. Additionally, this is the first report where CRISPR/Cas9/sgRNA-mediated genome editing was utilized to knockout a gene in *Nocardia* species. The two knockout clones grow well in LB or BHI medium just like the wild-type *N*. *cholesterolicum* NRRL5767. They can transform added OA to solely 10-HSA thus eliminating downstream separation from 10-KSA which has potential advantages in terms of reducing the costs and efforts. The potential industrial applications of these knockout mutants to transform OA and LA from acidified soapstock to the corresponding value-added HFAs will require further investigation.

## Supporting information

S1 Fig Amino acid sequence alignment of the NcOhy1, NcOhy2, and OhyA. The OhyA sequence is from *Elizabethkingia meningoseptica*. The putative conserved Rossmann fold is shown in blue and the putative FAD binding pocket in red.
(DOCX)

S2 Fig Amino acid sequence alignment of the Nc2o-ADH and Ml2o-ADH isolated from *M*. *luteus* WIUJH20. (DOCX)

S3 Fig Mass spectrum of the silylated 10-HSA. The y-axis is relative abundance of the ions referred to base peak (the mass of 331.2 Da is the base peak which has the greatest ion intensity) and the x-axis is mass-to-charge ratio (m/z). Each vertical line represents an ion having a specific m/z and the high of the vertical line indicates the relative abundance of the ion referred to the base peak.
(DOCX)

S4 Fig Mass spectrum of the silylated 10OH-12OD. The y-axis is relative abundance of the ions referred to base peak (the mass of 331.2 Da is the base peak which has the greatest ion intensity) and the x-axis is mass-to-charge ratio (m/z). Each vertical line represents an ion having a specific m/z and the high of the vertical line indicates the relative abundance of the ion referred to the base peak.
(DOCX)

S5 Fig Amino acid sequence alignment of the N-terminal 120 residues of Ohases from various *Nocardia* species. The sequences were obtained from a search of Ohases from *Nocardia* in Protein knowledgebase (https://www.uniprot.org/). The putative conserved Rossmann fold is shown in blue and the FAD binding pocket in red.
(DOCX)

S6 Fig Agarose gel electrophoresis of genomic DNA isolated from wild-type *N*. *cholesterolicum* NRRL5767, clone 1-p-11, and knockout mutants 1-3-17 & 2-3-51. Lane 1 is the wild-type *N*. *cholesterolicum* NRRL5767. Lane 2 is clone 1-p-11. Lanes 3 and 4 are knockout mutants 1-3-17 and 2-3-51, respectively. Lane 5 is the Hi-Lo DNA marker.
(DOCX)

S7 Fig Nucleotide sequence alignment of the 1.1 kb PCR fragment (amplified from the genomic DNA of 1-p-11 using primers 13+17) with the *Nc2o-ADH* coding sequence and its upstream. (DOCX)

S8 Fig Nucleotide sequence alignment of the 1.1 kb PCR fragments (1-3-17_PCR and 2-3-52_PCR) with the homologous sequences found in *N*. *cholesterolicum* NRRL5767 genome (NCNRRL5767$) and *Rhodococcus erythropolis* strain X5 genome (CP044284.1#). The fragments were amplified from the genomic DNA of knockout mutants 1-3-17 and 2-3-52 using primers 13+17. The NCNRRL5767$ sequence covers nucleotides 507572–506579. The CP044284.1# sequence covers nucleotides 65626–66619. This sequence encodes part of glutamate synthase large subunit.
(DOCX)

S1 Raw Images (PDF)
