# Supplementary material for: Knockout of secondary alcohol dehydrogenase in Nocardia cholesterolicum NRRL 5767 by CRISPR/Cas9 genome editing technology
Source: PLoS One. 2020 Mar 27;15(3):e0230915. doi: 10.1371/journal.pone.0230915 (PMC7101164; doi:10.1371/journal.pone.0230915)
Supplement: S1 Fig — The OhyA sequence is from Elizabethkingia meningoseptica. The putative conserved Rossmann fold is shown in blue and the putative FAD binding pocket in red. (DOCX) [file pone.0230915.s001.docx]

S1 Fig.

**NcOhy1 ---------------------------------------MY--YSSGNYEAFARPRKPDG 19**

**NcOhy2 ---------------------------------------------------------MSS 3**

**OhyA MNPITSKFDKVLNASSEYGHVNHEPDSSKEQQRNTPQKSMPFSDQIGNYQRN-KGIPVQS 59**

**..**

**NcOhy1 VDGKTAWFVGSGLASLAGAAFMIRDGQMAGNNITVLERLKLPGGALDGI----------- 68**

**NcOhy2 NLSHKAYMIGAGIGNLSAAVYLIRDGEWNGEDITIMGLD-MHG-ANDGESAATFQHQYGH 61**

**OhyA YDNSKIYIIGSGIAGMSAAYYFIRDGHVPAKNITFLEQLHIDGGSLDGA----------- 108**

**. . :::*:*:..::.* ::****. .::**.: : * : ****

**NcOhy1 --KEPEKGFVIRGGREM-EDHFECLWDLFRSVPSIEVED-ASVLDEFYWLNKDDPNYSLQ 124**

**NcOhy2 RELGNDAGFINRGGRMLNEETYENLWDILSAVPSLDNPG-KSVTDDILDFDHAHPTHDVA 120**

**OhyA --GNPTDGYIIRGGREM-DMTYENLWDMFQDIPALEMPAPYSVLDEYRLINDNDSNYSKA 165**

***:: **** : : :* ***:: :*::: ** *: ::. . .:.**

**NcOhy1 RVTERQGEDAHTDF---KFNLNSKAQKDIMKVF---LTPRSELENKRINEVFGK--EFLA 176**

**NcOhy2 RLIDRDGIRNKGENDYKHMQFDNKDRYLLTKLMTMPESDEAKLDDISIEQWFEDTPHFFT 180**

**OhyA RLINNKGEIK-DFS---KFGLNKMDQLAIIRLL---LKNKEELDDLTIEDYFSE--SFLK 216**

***: :..* :: ::. : : ::: . . :*:: *:: * . *:**

**NcOhy1 SNFWLYWRTMFAFEEWHSALEIKLYLHRFIHHIKGLPDLSTLKFTKYNQYESLVLPLYTW 236**

**NcOhy2 TNFWYMWETTFAFKRVSSAMELRRYMNRMILEFSRIQTLAGVTRSPYNQYESIILPMRTF 240**

**OhyA SNFWTFWRTMFAFENWHSLLELKLYMHRFLHAIDGLNDLSSLVFPKYNQYDTFVTPLRKF 276**

**:*** *.* ***:. * :*:: *::*:: :. : *: : ****:::: *: .:**

**NcOhy1 LLDQGVNFRFDTEVTDIDFDITGEV--KRAKRI--HWKSEGVLGGVDLDDGDLVLTTIGS 292**

**NcOhy2 LEGKGVKFVNELKITEFVFKDTPLRDEIIVTGLDYENVRTGEKGRIDVAEGDFVFDTNGS 300**

**OhyA LQEKGVNIHLNTLVKDLDIHINTEG--KVVEGI--ITEQDGKEVKIPVGKNDYVIVTTGS 332**

*** :**:: : :.:: :. . . : * : : ..* *: * ****

**NcOhy1 LTENSNNGDHHTPA-----KLDEGPAPAWDLWRRIATKDPAFGRPDVFGGHVAETKWESA 347**

**NcOhy2 ITDSSSIGDLDTPIVED-----MRYAPSALLWKQATEHFYDLGNPDKFFGDRAQSEWTSF 355**

**OhyA MTEDTFYGNNKTAPIIGIDNSTSGQSAGWKLWKNLAAKSEIFGKPEKFCSNIEKSAWESA 392**

**:*:.: *: .* : . **:. : : :*.*: * .. :: * ***

**NcOhy1 TVTTLDARIPEYIEKICKRDPFSGRVVTGGIVTARDSKWLMSWTVNRQPHFKQQPKDQIV 407**

**NcOhy2 TVTTSSHELINEISRITKQLP------GNALNTFVDSNVLLSIVVHHQPHYHAQKENEGV 409**

**OhyA TLTCKPSALIDKLKEYSVNDPYSGKTVTGGIITITDSNWLMSFTCNRQPHFPEQPDDVLV 452**

***:* : : :.. . * ..: * **: *:* . ::***: * .: ***

**NcOhy1 VWVYSLFVDVPGDYVDKPMQECTGEEITQEWLYHMGVPVEDI------PELAANAAKTVP 461**

**NcOhy2 FWGYCLFPRKDGDYVKKPFIEMTGREMLEETLGHLEALDESGALAARRQEITGSVVNSIP 469**

**OhyA LWVYALFMDKEGNYIKKTMLECTGDEILAELCYHLGIEDQ--------LENVQKNTIVRT 504**

**.* *.** *:*:.* : * ** *: * *: : * . . .**

**NcOhy1 VMMPYVTSFFMPRQAGDRPAVVPDGAVNFAFLGQFAETTRDCIFTTEYSVRTGMEAAYSL 521**

**NcOhy2 SHMPYASALFNRRAVGDRPLVVPKHSKNLAFISQFAELPFDMVFTEQYSVRCAQVAVYKF 529**

**OhyA AFMPYITSMFMPRAKGDRPRVVPEGCKNLGLVGQFVETNNDVVFTMESSVRTARIAVYKL 564**

***** :::* * **** ***. . *:.::.**.* * :** : *** . *.*.:**

**NcOhy1 LGIERGVPEV-FGSTYDIRKLLQATYFLNDKKEESVPLPKLLRRRLDKKLDENEIGQLLH 580**

**NcOhy2 LGIPEDKLTKMHHYEKDPKVLAKAAVTMFR------------------------------ 559**

**ohyA LNLNKQVPDI-NPLQYDIRHLLKAAKTLNDDKPFVG--EGLLRKVLKGTYF--------- 612**

***.: . * : * :*: :**

**NcOhy1 QYHILPRE--------------------------- 588**

**NcOhy2 ----------------------------------- 559**

**ohyA -EHVLPAGAAEEEEHESFIAEHVNKFREWVKGIRG 646**

**S1 Fig. Amino acid sequence alignment of the NcOhy1, NcOhy2, and OhyA.**

The OhyA sequence is from *Elizabethkingia meningoseptica*. The putative conserved Rossmann fold is shown in blue and the putative FAD binding pocket in red.
