# Supplementary figures and images for: Knockout of secondary alcohol dehydrogenase in Nocardia cholesterolicum NRRL 5767 by CRISPR/Cas9 genome editing technology
Source: PLoS One. 2020 Mar 27;15(3):e0230915. doi: 10.1371/journal.pone.0230915 (PMC7101164; doi:10.1371/journal.pone.0230915)

S2 Fig


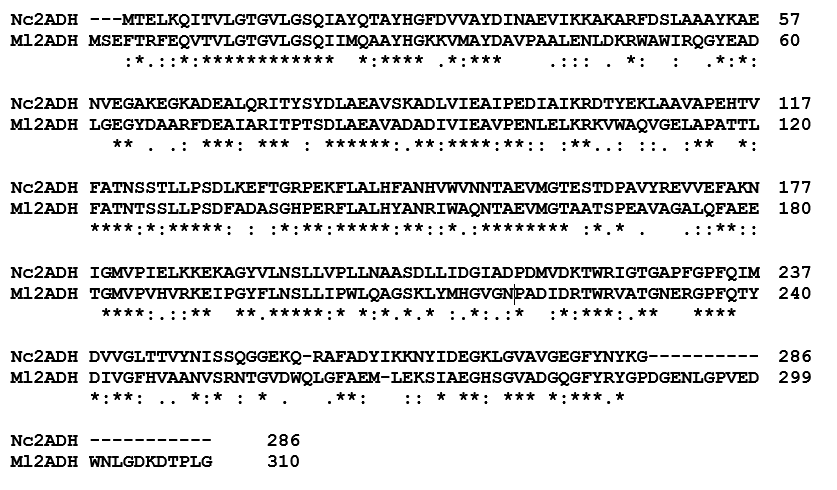


**S2 Fig. Amino acid sequence alignment of the Nc2o-ADH and Ml2o-ADH isolated from *M. luteus* WIUJH20.**

Supplement: S2 Fig — (DOCX) [file pone.0230915.s002.docx]
