# Supplementary material for: Knockout of secondary alcohol dehydrogenase in Nocardia cholesterolicum NRRL 5767 by CRISPR/Cas9 genome editing technology
Source: PLoS One. 2020 Mar 27;15(3):e0230915. doi: 10.1371/journal.pone.0230915 (PMC7101164; doi:10.1371/journal.pone.0230915)
Supplement: S4 Fig — The y-axis is relative abundance of the ions referred to base peak (the mass of 331.2 Da is the base peak which has the greatest ion intensity) and the x-axis is mass-to-charge ratio (m/z). Each vertical line represents an ion having a specific m/z and the high of the vertical line indicates the relative abundance of the ion referred to the base peak. (DOCX) [file pone.0230915.s004.docx]

S4 Fig


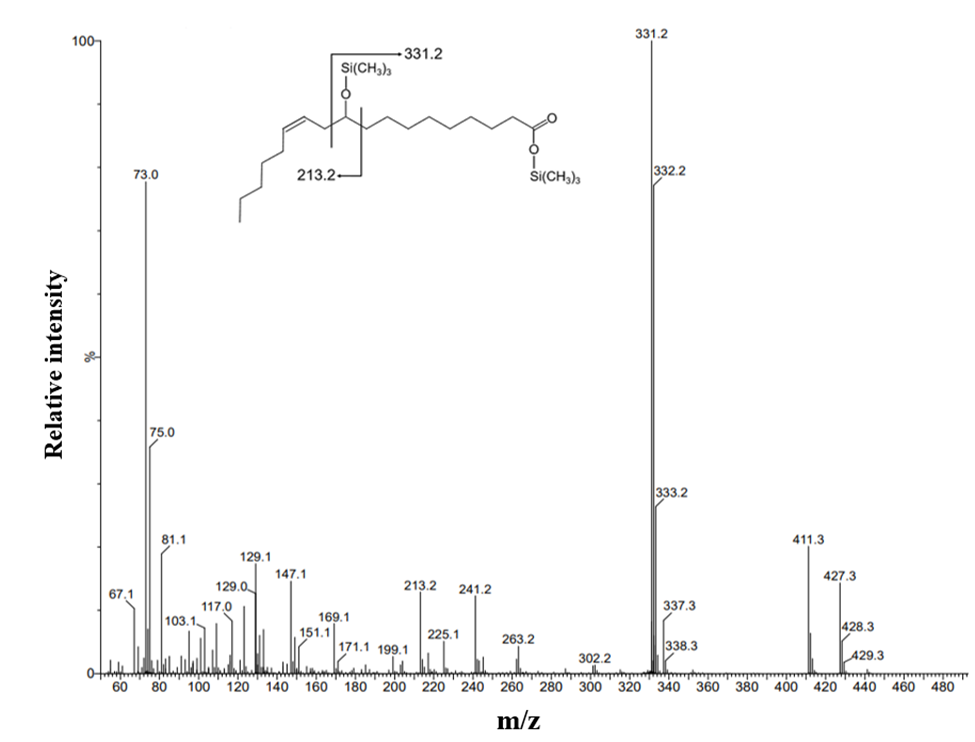


**S4 Fig. Mass spectrum of the Silylated 10OH-12OD.**

The y-axis is relative abundance of the ions referred to base peak (the mass of 331.2 Da is the base peak which has the greatest ion intensity) and the x-axis is mass-to-charge ratio (m/z). Each vertical line represents an ion having a specific m/z and the high of the vertical line indicates the relative abundance of the ion referred to the base peak.
