# Supplementary material for: Knockout of secondary alcohol dehydrogenase in Nocardia cholesterolicum NRRL 5767 by CRISPR/Cas9 genome editing technology
Source: PLoS One. 2020 Mar 27;15(3):e0230915. doi: 10.1371/journal.pone.0230915 (PMC7101164; doi:10.1371/journal.pone.0230915)
Supplement: S5 Fig — The sequences were obtained from a search of Ohases from Nocardia in Protein knowledgebase (https://www.uniprot.org/). The putative conserved Rossmann fold is shown in blue and the FAD binding pocket in red. (DOCX) [file pone.0230915.s005.docx]

S5 Fig.

**NcOhy1 MYYSSGNYEAFARPRKPDGVDGKTAWFVGSGLASLAGAAFMIRDGQMAGNNITVLERLKL 60**

**A0A386ZAF5 MYYSSGNYEAFARPRKPAGVEKKTAWFVGAGLASLSGAAFLIRDGQLPGNKITILEELKL 60**

**A0A1I9Z360 MYYSSGNYEAFARPRKPAGADGKTAWFVGSGLASLSGAAFLIRDGQMSGDKITVLEELKL 60**

**A0A0B8NND1 MYYSSGNYEAFARPRKPAGADGKTAWFVGSGLASLSGAAFLIRDGQMSGDKITVLEELKL 60**

**A0A318KA21 MYYSSGNYEAFARPRKPDGVEDKTAWFVGAGLASLSGAAFLIRDGQLPGEKITVFERLKL 60**

**A0A4P7GWZ8 MYYSSGNYEAFARPRKPEGVQDKTAWFVGAGLASLSGAAFLIRDGQMSGDKITVFERLKL 60**

**A0A511M7J8 MYYSSGNYEAFARPRKPEGVQDKTAWFVGAGLASLSGAAFLIRDGQMSGDKITVFERLKL 60**

**A0A1J0VV86 MYYSSGNYEAFARPRKPEGVDDKSAWFVGAGLASMSGAAFLIRDGQLSGDRITILERLPL 60**

**U5ED80 MYYSSGNYEAFARPRKPAGVDGKAAWFVGAGLASLSGAAFLIRDGQLAGDRITVLERLKL 60**

**A0A370GSU8 MYYSSGNYEAFARPRKPAGVDDKTAWFVGAGLASLSGAAFLIRDGRMPGNKITILEELRI 60**

**W5TAJ9 MYYSSGNYEAFARPRKPEGVDDKTAWFVGAGLASLSGAAFLIRDGQMSGDRITVLERLPL 60**

**A0A3M2L72 MYYSSGNYEAFARPRKPEGVDDKTAWFVGAGLASLSGAAFLIRDGQMSGDRITILERLKL 60**

**A0A2T2Z0L3 MYYSSGNYEAFARPRKPEGVDDKTAWFVGAGLASLSGAAFLIRDGQMSGDRITILERLKL 60**

**A0A1A0IVV4 MYYSSGNYEAFARPRKPEGVDDKTAWFVGAGLASLSGAAFLIRDGQMSGDRITILERLKL 60**

**A0A2S6A2X4 MYYSSGNYEAFARPRKPEGVDDKTAWFVGAGLASLSGAAFLIRDGQMSGDRITILERLKL 60**

**A0A378WLZ2 MYYSSGNYEAFARPRKPEGVDDKTAWFVGAGLASLSGAAFLIRDGQMSGDRITILERLKL 60**

**A0A2T2ZBI9 MYYSSGNYEAFARPRKPEGVDDKTAWFVGAGLASLSGAAFLIRDGQMSGDRITILERLKL 60**

******************* *.: *:*****:****::****:****:: *:.**::*.* :**

**NcOhy1 PGGALDGIKEPEKGFVIRGGREMEDHFECLWDLFRSVPSIEVEDASVLDEFYWLNKDDPN 120**

**A0A386ZAF5 PGGALDGIKEPEKGFVIRGGREMENHFECLWDLFRTVPSMEVEG-SVLDEFYWLNKDDPN 119**

**A0A1I9Z360 PGGALDGIKEPKKGFVIRGGREMENHFECLWDLYRTIPSIEIDGASVLDEFYWLNKDDPN 120**

**A0A0B8NND1 PGGALDGIKEPKKGFVIRGGREMENHFECLWDLYRTIPSIEIDGASVLDEFYWLNQDDPN 120**

**A0A318KA21 PGGALDGIKEPKKGFVIRGGREMENHFECLWDLFRSVPSIEIEGASVLDEFYWLNKDDPN 120**

**A0A4P7GWZ8 PGGALDGIKEPKKGFVIRGGREMENHFECLWDLFRSVPSMEIDGASVLDEFYWLNKDDPN 120**

**A0A511M7J8 PGGALDGIKEPKKGFVIRGGREMENHFECLWDLFRSVPSMEIDGASVLDEFYWLNKDDPN 120**

**A0A1J0VV86 PGGALDGIKKPKKGFVIRGGREMENHFECLWDLFRTVPSLEIEDASVLDEFYWLNKDDPN 120**

**U5ED80 PGGALDGIREPKKGFVIRGGREMENHFECLWDLYRSVPSIEIEGASVLDEFYWLNKDDPN 120**

**A0A370GSU8 PGGALDGIREPKKGFVIRGGREMENHFECLWDLFRSVPSLEIDDASVLDEFYWLNKDDPN 120**

**W5TAJ9 PGGALDGIKKPTKGFVIRGGREMENHFECLWDLFRSIPSIEISGASVLDEFYWLNKDDPN 120**

**A0A3M2L72 PGGALDGIKEPAKGFVIRGGREMENHFECLWDLFRSVPSIEIEGASVLDEFYWLNKDDPN 120**

**A0A2T2Z0L3 PGGALDGIKEPAKGFVIRGGREMENHFECLWDLFRSVPSIEIEGASVLDEFYWLNKDDPN 120**

**A0A1A0IVV4 PGGALDGIKEPAKGFVIRGGREMENHFECLWDLFRSVPSIEIEGASVLDEFYWLNKDDPN 120**

**A0A2S6A2X4 PGGALDGIKEPAKGFVIRGGREMENHFECLWDLFRSVPSIEIEGASVLDEFYWLNKDDPN 120**

**A0A378WLZ2 PGGALDGIKEPAKGFVIRGGREMENHFECLWDLFRSVPSIEIEGASVLDEFYWLNKDDPN 120**

**A0A2T2ZBI9 PGGALDGIKEPAKGFVIRGGREMENHFECLWDLFRSVPSIEIEGASVLDEFYWLNKDDPN 120**

**********::* ************:********:*::**:*:.. **********:******

**S5 Fig. Amino acid sequence alignment of the N-terminal 120 residues of Ohases from various *Nocardia* species.**

The sequences were obtained from a search of Ohases from *Nocardia* in Protein knowledgebase (<https://www.uniprot.org/>). The putative conserved Rossmann fold is shown in blue and the FAD binding pocket in red.
