# Supplementary material for: Knockout of secondary alcohol dehydrogenase in Nocardia cholesterolicum NRRL 5767 by CRISPR/Cas9 genome editing technology
Source: PLoS One. 2020 Mar 27;15(3):e0230915. doi: 10.1371/journal.pone.0230915 (PMC7101164; doi:10.1371/journal.pone.0230915)
Supplement: S6 Fig — Lane 1 is the wild-type N. cholesterolicum NRRL5767. Lane 2 is clone 1-p-11. Lanes 3 and 4 are knockout mutants 1-3-17 and 2-3-51, respectively. Lane 5 is the Hi-Lo DNA marker. (DOCX) [file pone.0230915.s006.docx]

S6 Fig


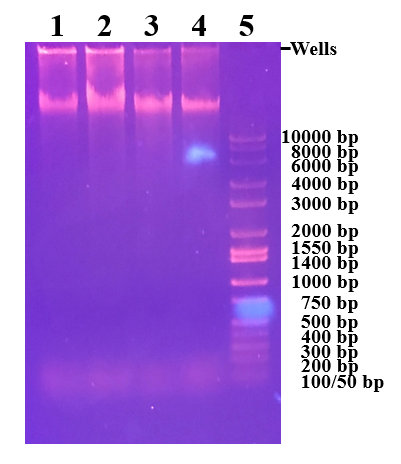


**S6 Fig. Agarose gel electrophoresis of genomic DNA isolated from wild-type** ***N. cholesterolicum* NRRL5767, clone 1-p-11, and knockout mutants 1-3-17 & 2-3-51.**

Lane 1 is the wild-type *N. cholesterolicum* NRRL5767. Lane 2 is clone 1-p-11. Lanes 3 and 4 are knockout mutants 1-3-17 and 2-3-51, respectively. Lane 5 is the Hi-Lo DNA marker.
