# Supplementary material for: Knockout of secondary alcohol dehydrogenase in Nocardia cholesterolicum NRRL 5767 by CRISPR/Cas9 genome editing technology
Source: PLoS One. 2020 Mar 27;15(3):e0230915. doi: 10.1371/journal.pone.0230915 (PMC7101164; doi:10.1371/journal.pone.0230915)
Supplement: S7 Fig — (DOCX) [file pone.0230915.s007.docx]

S7 Fig

**-265&Nc2-ADH GAGCCCAGCGAATGTTTCCGGGCCGAAGGTCTGGAACTCTCGCAGCGCCTGCAAACGCGG 60**

**1-p-11_PCR -------------------------------------TCTCGCAGCGC-TGCAA-CGCGG** **21**

************* ***** *******

**-265&Nc2-ADH ATTGCGCAAATCTTGCTCGAGGGGCACCCCAACCACACCACCCGAACCATCACTGCAGCG 120**

**1-p-11_PCR ATTGCGCAAATCTTGCTCGAGGGGCACCCCAACCACACCACCCGAACCATCACTGCAGCG 81**

****************************************************************

**-265&Nc2-ADH CGAACGATTTCCAACGTGCTGCACTTCGAAATAGCCCGCGCGGCATTGGACCGGATCACG 180**

**1-p-11_PCR CGAACGATTTCCAACGTGCTGCACTTCGAAATAGCCCGCGCGGCATTGGACCGGATCACG 141**

****************************************************************

**-265&Nc2-ADH GCTGACGAACTCACGAACATCGTTGCCGCACAAGTTCGATTGACGCTTGCCGGACTACCC 240**

**1-p-11_PCR GCTGACGAACTCACGAACATCGTTGCCGCACAAGTTCGATTGACGCTTGCCGGACTACCC 201**

****************************************************************

**-265&Nc2-ADH ACCGAAAAGGATTGAGGATCGAAAATGACTGAACTGAAGCAGATCACCGTTCTGGGTACC 300**

**1-p-11_PCR ACCGAAAAGGATTGAGGATCGAAAATGACTGAACTGAAGCAGATCACCGTTCTGGGTACC 261**

****************************************************************

**-265&Nc2-ADH GGAGTTCTCGGCTCGCAGATCGCCTATCAGACCGCCTATCACGGATTCGACGTCGTCGCG 360**

**1-p-11_PCR GGAGTTCTCGGCTCGCAGATCGCCTATCAGACCGCCTATCACGGATTCGACGTCGTCGCG 321**

****************************************************************

**-265&Nc2-ADH TACGACATCAACGCCGAGGTCATCAAGAAGGCCAAGGCTCGGTTCGACTCGTTGGCCGCG 420**

**1-p-11_PCR TACGACATCAACGCCGAGGTCATCAAGAAGGCCAAGGCTCGGTTCGACTCGTTGGCCGCG 381**

****************************************************************

**-265&Nc2-ADH GCCTACAAGGCCGAAAACGTCGAGGGCGCCAAGGAAGGCAAGGCCGACGAAGCGCTGCAA 480**

**1-p-11_PCR GCCTACAAGGCCGAAAACGTCGAGGGCGCCAAGGAAGGCAAGGCCGACGAAGCGCTGCAA 441**

****************************************************************

**-265&Nc2-ADH CGTATTACGTACTCGTACGACCTAGCCGAAGCCGTGTCCAAGGCCGATCTTGTCATCGAG 540**

**1-p-11_PCR CGTATTACGTACTCGTACGACCTAGCCGAAGCCGTGTCCAAGGCCGATCTTGTCATCGAG 501**

****************************************************************

**-265&Nc2-ADH GCAATTCCCGAGGACATCGCCATCAAGCGCGACACCTACGAGAAGCTCGCCGCAGTAGCT 600**

**1-p-11_PCR GCAATTCCCGAGGACATCGCCATCAAGCGCGACACCTACGAGAAGCTCGCCGCAGTAGCT 561**

****************************************************************

**-265&Nc2-ADH CCTGAGCACACGGTGTTCGCAACCAACTCCTCGACGCTTCTGCCGAGCGACCTCAAGGAG 660**

**1-p-11_PCR CCTGAGCACACGGTGTTCGCAACCAACTCCTCGACGCTTCTGCCGAGCGACCTCAAGGAG 621**

****************************************************************

**-265&Nc2-ADH TTCACCGGCCGCCCCGAGAAGTTCCTCGCACTGCACTTCGCAAATCACGTGTGGGTCAAC 720**

**1-p-11_PCR TTCACCGGCCGCCCCGAGAAGTTCCTCGCACTGCACTTCGCAAATCACGTGTGGGTCAAC 681**

****************************************************************

**-265&Nc2-ADH AACACTGCCGAGGTCATGGGCACCGAGTCCACCGACCCCGCCGTGTACCGCGAGGTCGTC 780**

**1-p-11_PCR AACACTGCCGAGGTCATGGGCACCGAGTCCACCGACCCCGCCGTGTACCGCGAGGTCGTC 741**

****************************************************************

**-265&Nc2-ADH GAATTCGCGAAGAACATCGGCATGGTGCCGATCGAACTCAAGAAGGAGAAGGCGGGCTAC 840**

**1-p-11_PCR GAATTCGCGAAGAACATCGGCATGGTGCCGATCGAACTCAAGAAGGAGAAGGCGGGCTAC 801**

****************************************************************

**-265&Nc2-ADH GTACTCAACTCGCTCCTGGTCCCGCTCCTCAATGCAGCATCCGACCTGCTGATCGACGGC 900**

**1-p-11_PCR GTACTCAACTCGCTCCTGGTCCCGCTCCTCAATGCAGCATCCGACCTGCTGATCGACGGC 861**

****************************************************************

**-265&Nc2-ADH ATCGCCGATCCCGACATGGTCGACAAGACGTGGCGTATCGGCACCGGAGCCCCGTTCGGC 960**

**1-p-11_PCR ATCGCCGATCCCGACATGGTCGACAAGACGTGGCGTATCGGCACCGGAGCCCCGTTCGGC 921**

****************************************************************

**-265&Nc2-ADH CCCTTCCAGATCATGGATGTCGTCGGGTTGACCACCGTCTACAACATCTCCTCCCAGGGC 1020**

**1-p-11_PCR CCCTTCCAGATCATGGATGTCGTCGGGTTGACCACCGTCTACAACATCTCCTCCCAGGGC 981**

****************************************************************

**-265&Nc2-ADH GGCGAGAAGCAGCGCGCGTTCGCCGACTACATCAAGAAGAACTACATCGACGAAGGCAAG 1080**

**1-p-11_PCR GGCGAGAAGCAGCGCGCGTTCGCCGACTACATCAAGAAGAACTACATCGACGAAT----- 1036**

**********************************************************

**-265&Nc2-ADH CTCGGCGTCGCTGTCGGCGAGGGCTTCTACAACTACAAAGGCTGA 1125**

**1-p-11_PCR --------------------------------------------- 1036**

**S7 Fig. Nucleotide sequence alignment of the 1.1 kb PCR fragment (amplified from the genomic DNA of 1-p-11 using primers 13+17) with the *Nc2^o^-ADH* coding sequence and its upstream.**
