# Supplementary material for: Knockout of secondary alcohol dehydrogenase in Nocardia cholesterolicum NRRL 5767 by CRISPR/Cas9 genome editing technology
Source: PLoS One. 2020 Mar 27;15(3):e0230915. doi: 10.1371/journal.pone.0230915 (PMC7101164; doi:10.1371/journal.pone.0230915)
Supplement: S8 Fig — The fragments were amplified from the genomic DNA of knockout mutants 1-3-17 and 2-3-52 using primers 13+17. The NCNRRL5767$ sequence covers nucleotides 507572–506579. The CP044284.1# sequence covers nucleotides 65626–66619. This sequence encodes part of glutamate synthase large subunit. (DOCX) [file pone.0230915.s008.docx]

S8 Fig

**1-3-17_PCR GTATCCTGTGAATCGGAACTTCTCTGCACAGACGCAGCTCCGAAATCTGTCGAGGGGCCT 60**

**2-3-52_PCR GTATCCTGTGAATCGGAACTTCTCTGCACAGACGCAGCTCCGAAATCTGTCGAGGGGCCT 60**

**NCNRRL5767$ GTATCCTGTGAATCGGAACTTCTCTGCACAGACGCAGCTCCGAAATCTGTCGAGGGGCCT 60**

**CP044284.1# GTATCCTGTGAATCGGAACTTCTCTGCACAGACGCAGCTCCGAAATCTGTCGAGGGGCCT 60**

****************************************************************

**1-3-17_PCR TCTGGCTCCCTGCATCGCCGCAGTTCAGAGCAATTATAGGGCTACCTTAGTTGCGCCCGT 120**

**2-3-52_PCR TCTGGCTCCCTGCATCGCCGCAGTTCAGAGCAATTATAGGGCTACCTTAGTTGCGCCCGT 120**

**NCNRRL5767$ TCTGGCTCCCTGCATCGCCGCAGTTCAGAGCAATTATAGGGCTACCTTAGTTGCGCCCGT 120**

**CP044284.1# TCTGGCTCCCTGCATCGCCGCAGTTCAGAGCAATTATAGGGCTACCTTAGTTGCGCCCGT 120**

****************************************************************

**1-3-17_PCR GCCGGGGTTCTGAATTATCGTTTTCGTTACACGGTTGACTCACAAAAGTTTCAACCGACC 180**

**2-3-52_PCR GCCGGGGTTCTGAATTATCGTTTTCGTTACACGGTTGACTCACAAAAGTTTCAACCGACC 180**

**NCNRRL5767$ GCCGGGGTTCTGAATTATCGTTTTCGTTACACGGTTGACTCACAAAAGTTTCAACCGACC 180**

**CP044284.1# GCCGGGGTTCTGAATTATCGTTTTCGTTACACGGTTGACTCACAAAAGTTTCAACCGACC 180**

****************************************************************

**1-3-17_PCR GAAGACTCACGTCGCCGGGTGCCAGAACGCACACCATCGCGTCTGCGCAGGGCGGCTACT 240**

**2-3-52_PCR GAAGACTCACGTCGCCGGGTGCCAGAACGCACACCATCGCGTCTGCGCAGGGCGGCTACT 240**

**NCNRRL5767$ GAAGACTCACGTCGCCGGGTGCCAGAACGCACACCATCGCGTCTGCGCAGGGCGGCTACT 240**

**CP044284.1# GAAGACTCACGTCGCCGGGTGCCAGAACGCACACCATCGCGTCTGCGCAGGGCGGCTACT 240**

****************************************************************

**1-3-17_PCR TCGGTGGTACCGAACACATCGTCGTGTCGGTGCTTTGAAGGACTTGCATTGCAAGTTGAT 300**

**2-3-52_PCR TCGGTGGTACCGAACACATCGTCGTGTCGGTGCTTTGAAGGACTTGCATTGCAAGTTGAT 300**

**NCNRRL5767$ TCGGTGGTACCGAACACATCGTCGTGTCGGTGCTTTGAAGGACTTGCATTGCAAGTTGAT 300**

**CP044284.1# TCGGTGGTACCGAACACATCGTCGTGTCGGTGCTTTGAAGGACTTGCATTGCAAGTTGAT 300**

****************************************************************

**1-3-17_PCR GGCCATCAGCGCTGCTGGTCGTCGGTGCATCTTTATGGCATACGAAGGCTGGGTATGAAG 360**

**2-3-52_PCR GGCCATCAGCGCTGCTGGTCGTCGGTGCATCTTTATGGCATACGAAGGCTGGGTATGAAG 360**

**NCNRRL5767$ GGCCATCAGCGCTGCTGGTCGTCGGTGCATCTTTATGGCATACGAAGGCTGGGTATGAAG 360**

**CP044284.1# GGCCATCAGCGCTGCTGGTCGTCGGTGCATCTTTATGGCATACGAAGGCTGGGTATGAAG 360**

****************************************************************

**1-3-17_PCR CAACTTCCAGGCCCCCAGGGCCTCTATCACCCGTCCAATGAGCACGACTCCTGTGGAGTC 420**

**2-3-52_PCR CAACTTCCAGGCCCCCAGGGCCTCTATCACCCGTCCAATGAGCACGACTCCTGTGGAGTC 420**

**NCNRRL5767$ CAACTTCCAGGCCCCCAGGGCCTCTATCACCCGTCCAATGAGCACGACTCCTGTGGAGTC 420**

**CP044284.1# CAACTTCCAGGCCCCCAGGGCCTCTATCACCCGTCCAATGAGCACGACTCCTGTGGAGTC 420**

****************************************************************

**1-3-17_PCR GCATTTGTTGTCGACATGCATGGTCGCCGCAGCAGGGACATCGTCGAAAAGGCGATCACC 480**

**2-3-52_PCR GCATTTGTTGTCGACATGCATGGTCGCCGCAGCAGGGACATCGTCGAAAAGGCGATCACC 480**

**NCNRRL5767$ GCATTTGTTGTCGACATGCATGGTCGCCGCAGCAGGGACATCGTCGAAAAGGCGATCACC 480**

**CP044284.1# GCATTTGTTGTCGACATGCATGGTCGCCGCAGCAGGGACATCGTCGAAAAGGCGATCACC 480**

****************************************************************

**1-3-17_PCR GCTCTGGTCAACCTCGAGCATCGCGGCGCTGCCGGATCAGAACCCAACACGGGTGACGGT 540**

**2-3-52_PCR GCTCTGGTCAACCTCGAGCATCGCGGCGCTGCCGGATCAGAACCCAACACGGGTGACGGT 540**

**NCNRRL5767$ GCTCTGGTCAACCTCGAGCATCGCGGCGCTGCCGGATCAGAACCCAACACGGGTGACGGT 540**

**CP044284.1# GCTCTGGTCAACCTCGAGCATCGCGGCGCTGCCGGATCAGAACCCAACACGGGTGACGGT 540**

****************************************************************

**1-3-17_PCR GCGGGCATTCTGCTGCAGGTTCCCGACAAGTTCTTCCGAGCTGTCGTGGACTTCGCGCTG 600**

**2-3-52_PCR GCGGGCATTCTGCTGCAGGTTCCCGACAAGTTCTTCCGAGCTGTCGTGGACTTCGCGCTG 600**

**NCNRRL5767$ GCGGGCATTCTGCTGCAGGTTCCCGACAAGTTCTTCCGAGCTGTCGTGGACTTCGCGCTG 600**

**CP044284.1# GCGGGCATTCTGCTGCAGGTTCCCGACAAGTTCTTCCGAGCTGTCGTGGACTTCGCGCTG 600**

****************************************************************

**1-3-17_PCR CCCGCCGAAGGCGCGTACGCAACCGGTATCGCATTCCTCCCGCAGGGCGATGCCGATGCA 660**

**2-3-52_PCR CCCGCCGAAGGCGCGTACGCAACCGGTATCGCATTCCTCCCGCAGGGCGATGCCGATGCA 660**

**NCNRRL5767$ CCCGCCGAAGGCGCGTACGCAACCGGTATCGCATTCCTCCCGCAGGGCGATGCCGATGCA 660**

**CP044284.1# CCCGCCGAAGGCGCGTACGCAACCGGTATCGCATTCCTCCCGCAGGGCGATGCCGATGCA 660**

****************************************************************

**1-3-17_PCR CACGAGGCTGCTGCTGCAGTCGAGAAGATCGTCGTCGAAGAGGGCCTGAAGGTCCTCGGC 720**

**2-3-52_PCR CACGAGGCTGCTGCTGCAGTCGAGAAGATCGTCGTCGAAGAGGGCCTGAAGGTTCTCGGC 720**

**NCNRRL5767$ CACGAGGCTGCTGCTGCAGTCGAGAAGATCGTCGTCGAAGAGGGCCTGAAGGTTCTCGGC 720**

**CP044284.1# CACGAGGCTGCTGCTGCAGTCGAGAAGATCGTCGTCGAAGAGGGCCTGAAGGTTCTCGGC 720**

******************************************************* ********

**1-3-17_PCR TGGCGCGAGGTCGGAACCGACGACTCCTCGCTGGGCGCATTGGCGCGCGACGCGATGCCG 780**

**2-3-52_PCR TGGCGCGAGGTCGGAACCGACGACTCCTCGCTGGGCGCATTGGCGCGCGACGCGATGCCG 780**

**NCNRRL5767$ TGGCGCGAGGTCGGAACCGACGACTCCTCGCTGGGCGCATTGGCGCGCGACGCGATGCCG 780**

**CP044284.1# TGGCGCGAGGTCGGAACCGACGACTCCTCGCTGGGCGCACTGGCGCGCGACGCGATGCCG 780**

***************************************** **********************

**1-3-17_PCR ACGTTCCGTCAGATCTTCATCGGCTCCGAGGGTGACAAGTACACCGGCATGGATCTCGAG 840**

**2-3-52_PCR ACGTTCCGTCAGATCTTCATCGGCTCCGAGGGTGACAAGTACACCGGCATGGATCTCGAG 840**

**NCNRRL5767$ ACGTTCCGTCAGATCTTCATCGGCTCCGAGGGTGACAAGTACACCGGCATGGATCTCGAG 840**

**CP044284.1# ACGTTCCGTCAGATCTTCATCGGCTCCGAGGGTGACAAGTACACCGGCATGGATCTCGAG 840**

****************************************************************

**1-3-17_PCR CGTCGTGCATACGTGGTGCGTAAGCGCACCGAGCACGAACTCGGCTCCGAAGGCGCCGGC 900**

**2-3-52_PCR CGTCGTGCATACGTGGTGCGTAAGCGCACCGAGCACGAACTCGGCTCCGAAGGCGCCGGC 900**

**NCNRRL5767$ CGTCGTGCATACGTGGTGCGTAAGCGCACCGAGCACGAACTCGGCTCCGAAGGCGCCGGC 900**

**CP044284.1# CGTCGTGCATACGTGGTGCGTAAGCGCACCGAGCACGAACTCGGCTCCGAAGGCGCCGGC 900**

****************************************************************

**1-3-17_PCR AAGGGCGGCCCGGGTAGCGAGACCGTGTACTTCCCCAGCCTGTCCGGCCAGACGTTCGTC 960**

**2-3-52_PCR AAGGGCGGCCCGGGTAGCGAGACCGTGTACTTCCCCAGCCTGTCCGGCCAGACGTTCGTC 960**

**NCNRRL5767$ AAGGGCGGCCCGGGTAGCGAGACCGTGTACTTCCCCAGCCTGTCCGGCCAGACGTTCGTC 960**

**CP044284.1# AAGGGTGGCCCGGGTAGCGAGACCGTGTACTTCCCCAGCCTGTCCGGTCAGACGTTCGTC 960**

******* ***************************************** **************

**1-3-17_PCR TACAAGGGCATGCTCACCACCCCGCAGCTCA--- 991**

**2-3-52_PCR TA-------------------------------- 962**

**NCNRRL5767$ TACAAGGGCATGCTCACCACCCCGCAGCTCAAGG 994**

**CP044284.1# TACAAGGGCATGCTCACCACCCCGCAGCTCAAGG 994**

******

**S8 Fig. Nucleotide sequence alignment of the 1.1 kb PCR fragments (1-3-17_PCR and 2-3-52_PCR) with the homologous sequences found in *N. cholesterolicum* NRRL5767 genome (NCNRRL5767$) and *Rhodococcus erythropolis* strain X5 genome (CP044284.1#).**

The fragments were amplified from the genomic DNA of knockout mutants 1-3-17 and 2-3-52 using primers 13+17. The NCNRRL5767$ sequence covers nucleotides 507572-506579. The CP044284.1# sequence covers nucleotides 65626-66619. This sequence encodes part of glutamate synthase large subunit.
