# Supplementary material for: Knockout of secondary alcohol dehydrogenase in Nocardia cholesterolicum NRRL 5767 by CRISPR/Cas9 genome editing technology
Source: PLoS One. 2020 Mar 27;15(3):e0230915. doi: 10.1371/journal.pone.0230915 (PMC7101164; doi:10.1371/journal.pone.0230915)

Fig 1

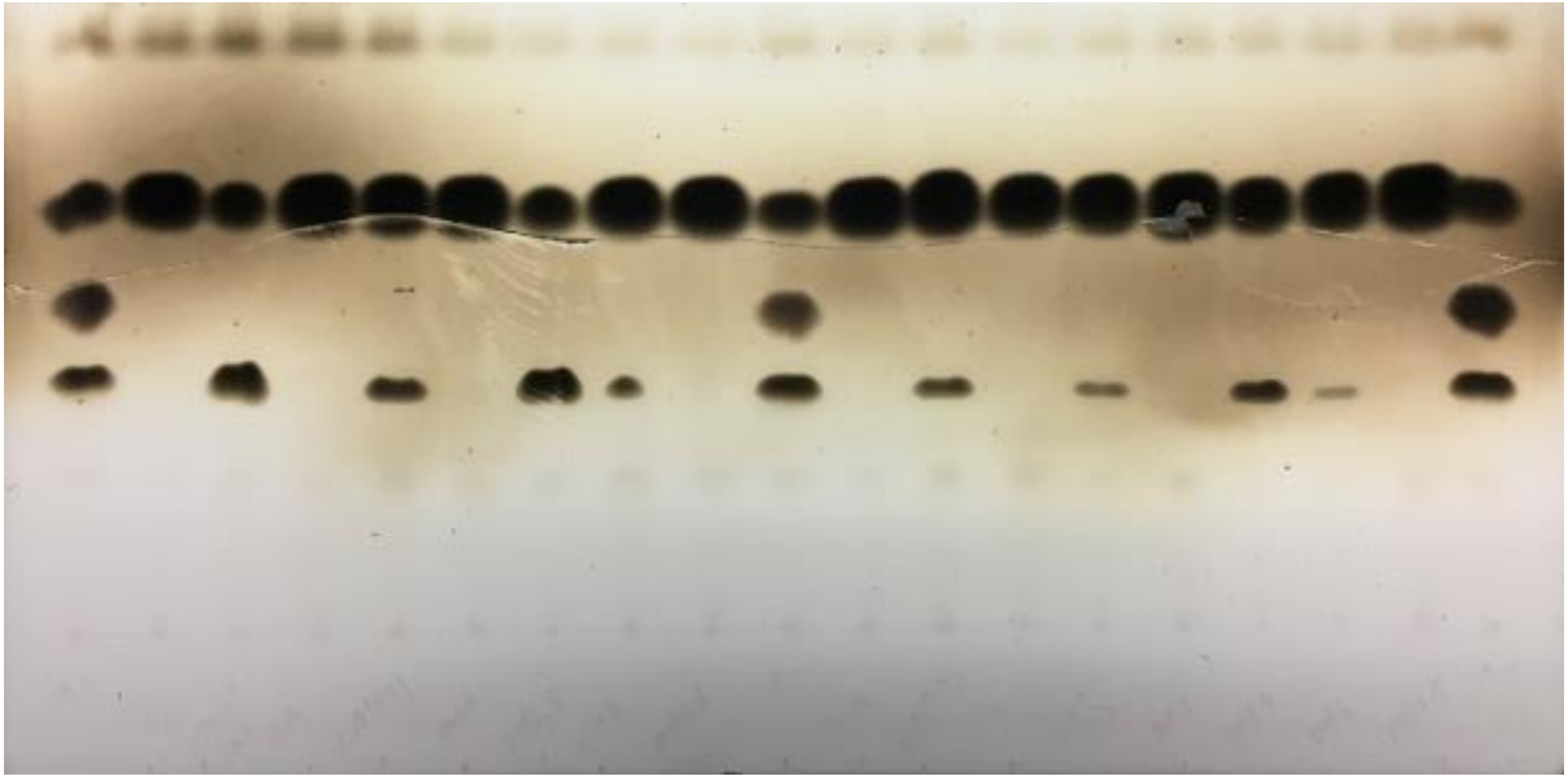

Fig 3

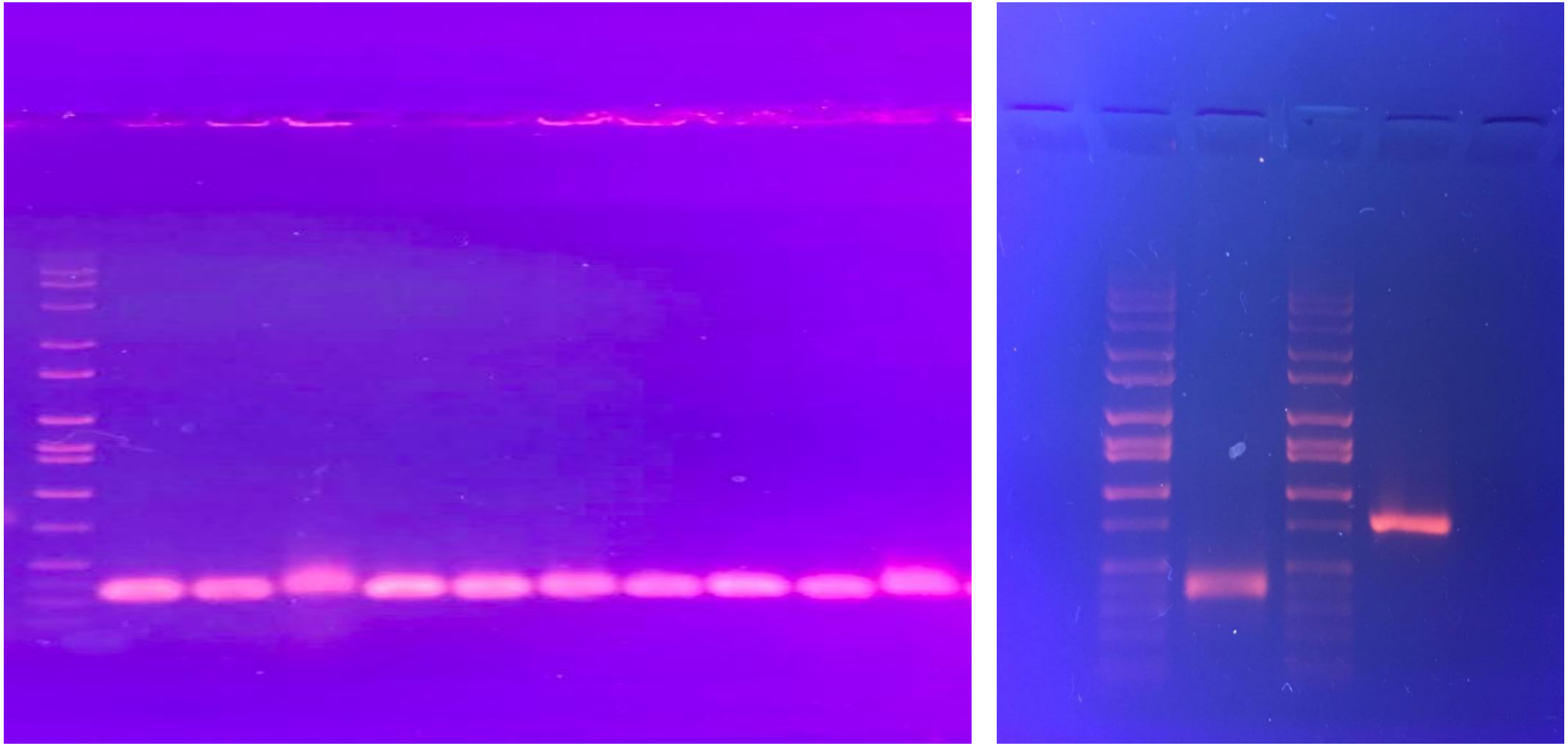

Fig 4 panel A

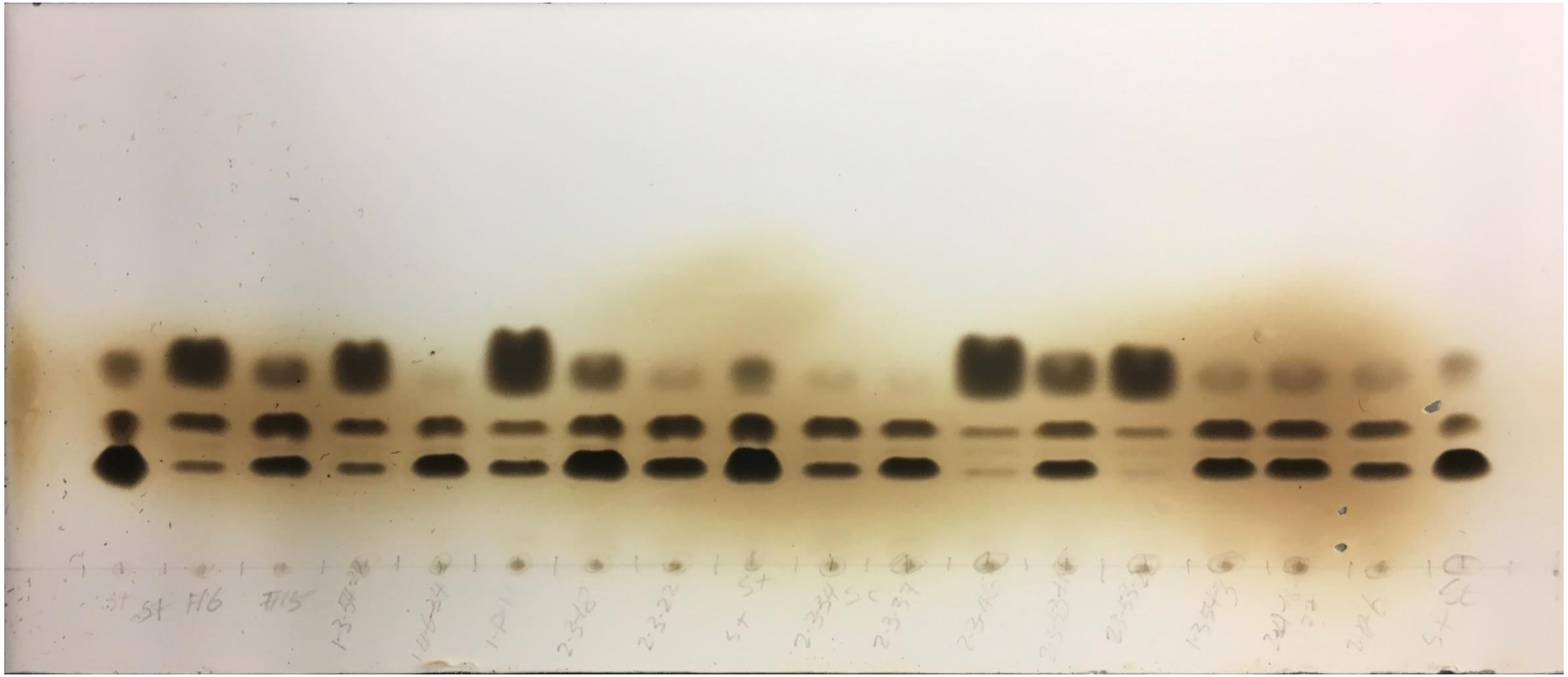

Fig 4 panel B

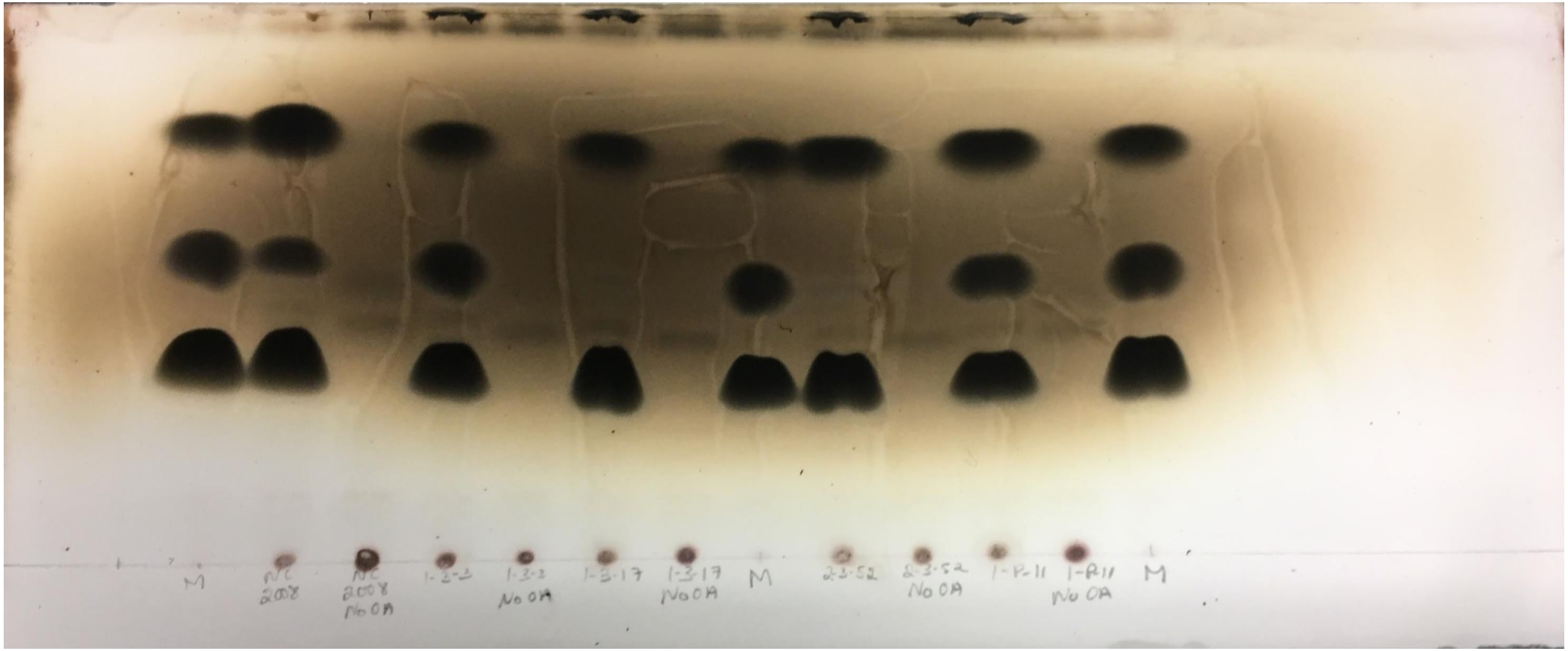
M
NC 2008
NC 2008 No OA
1-3-3
1-3-3 No OA
1-3-17
1-3-17 No OA
M
2-3-52
2-3-52 No OA
1-R-11
1-R-11 No OA
M

Fig 5

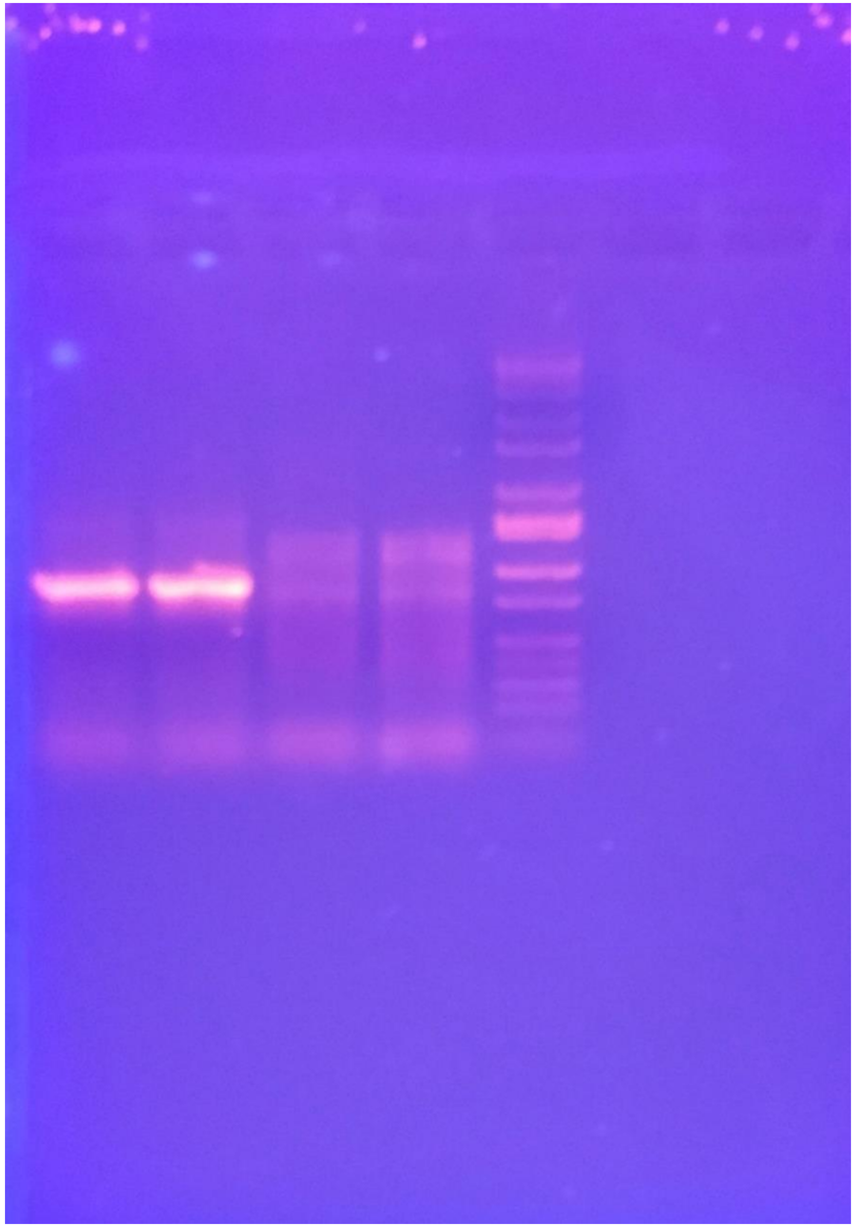

Fig 6, panel B

Wild type
1-p-11

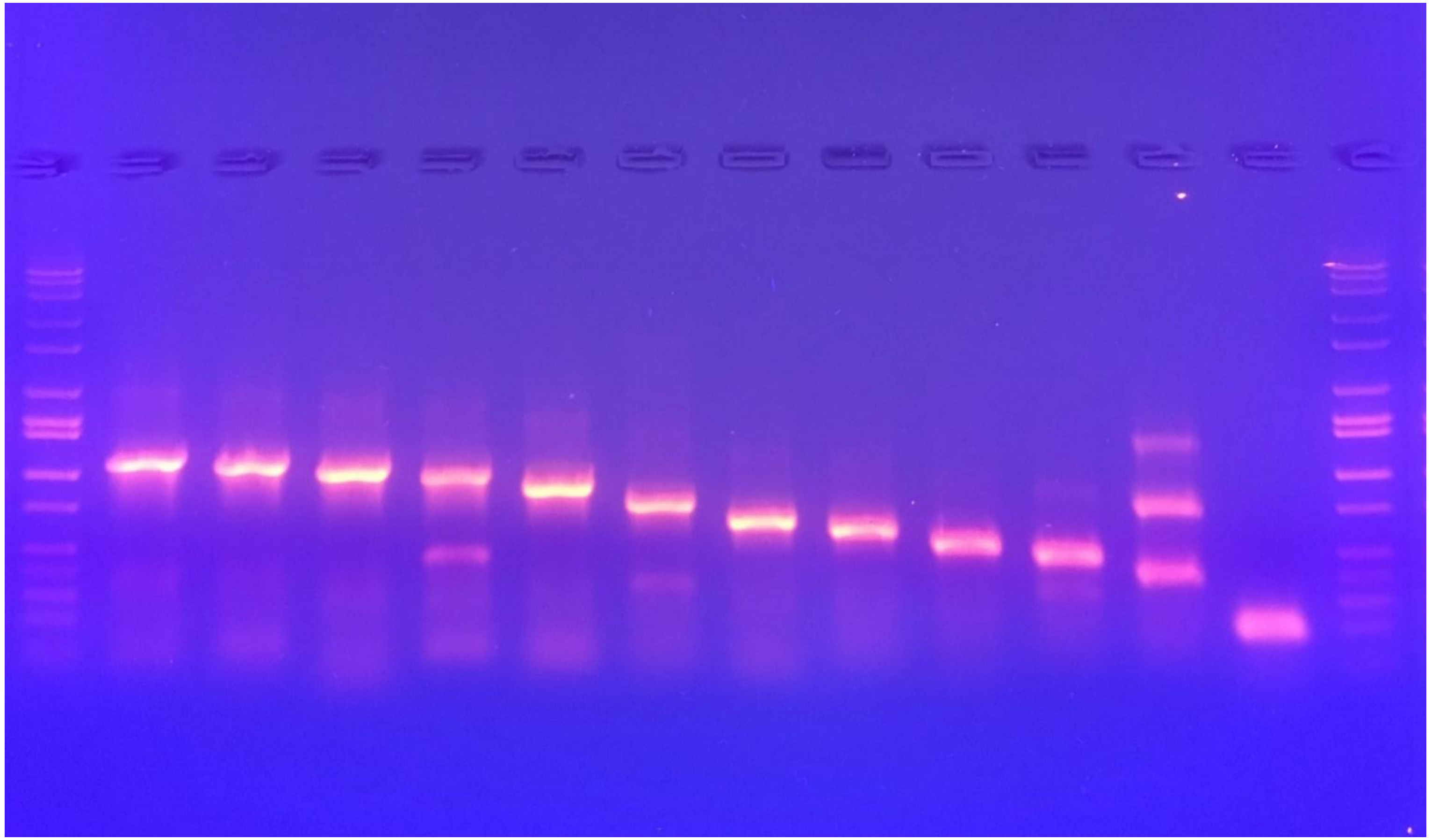

Fig 6, panel B

Knockout mutant
1-3-17

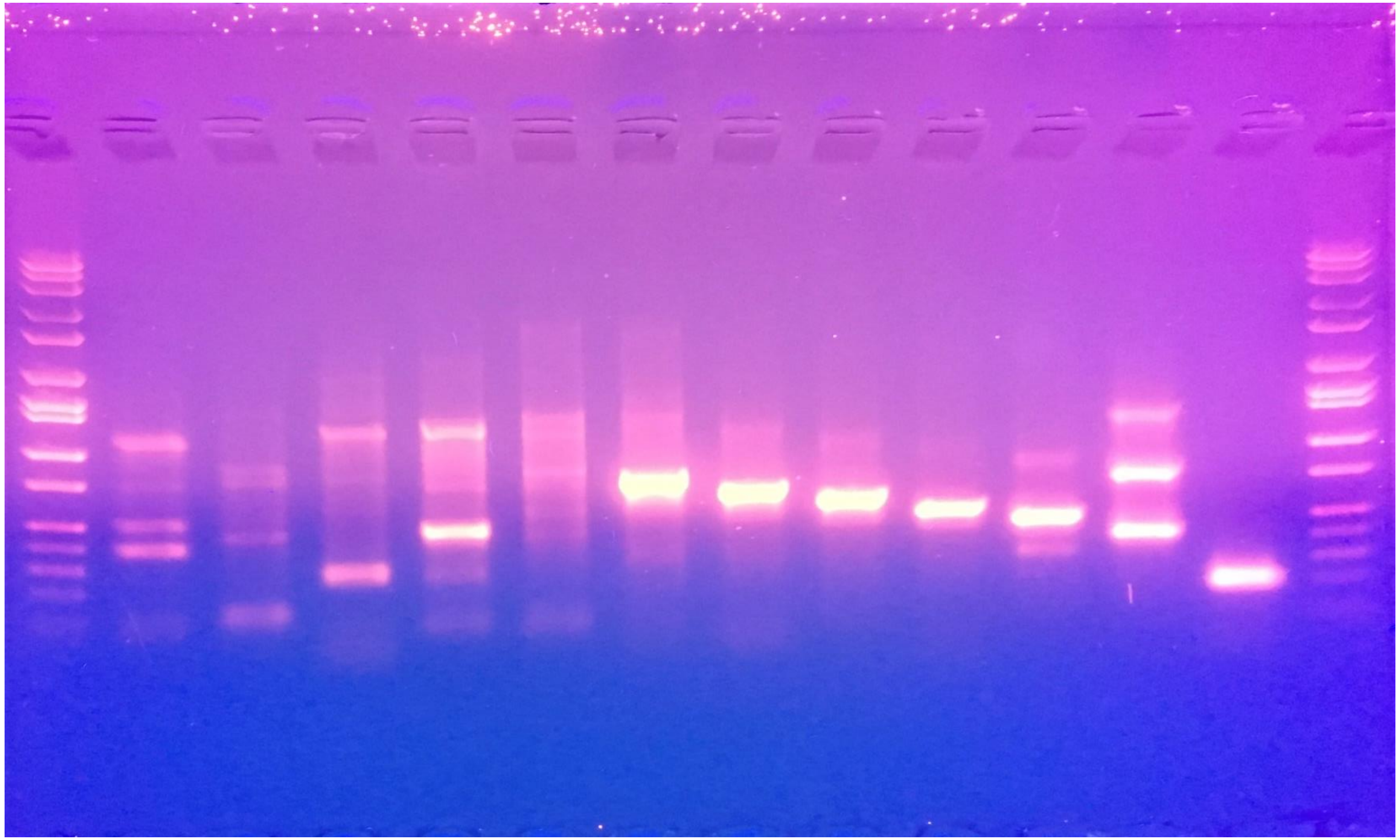

Fig 6, panel B

Knockout mutant 2-3-52

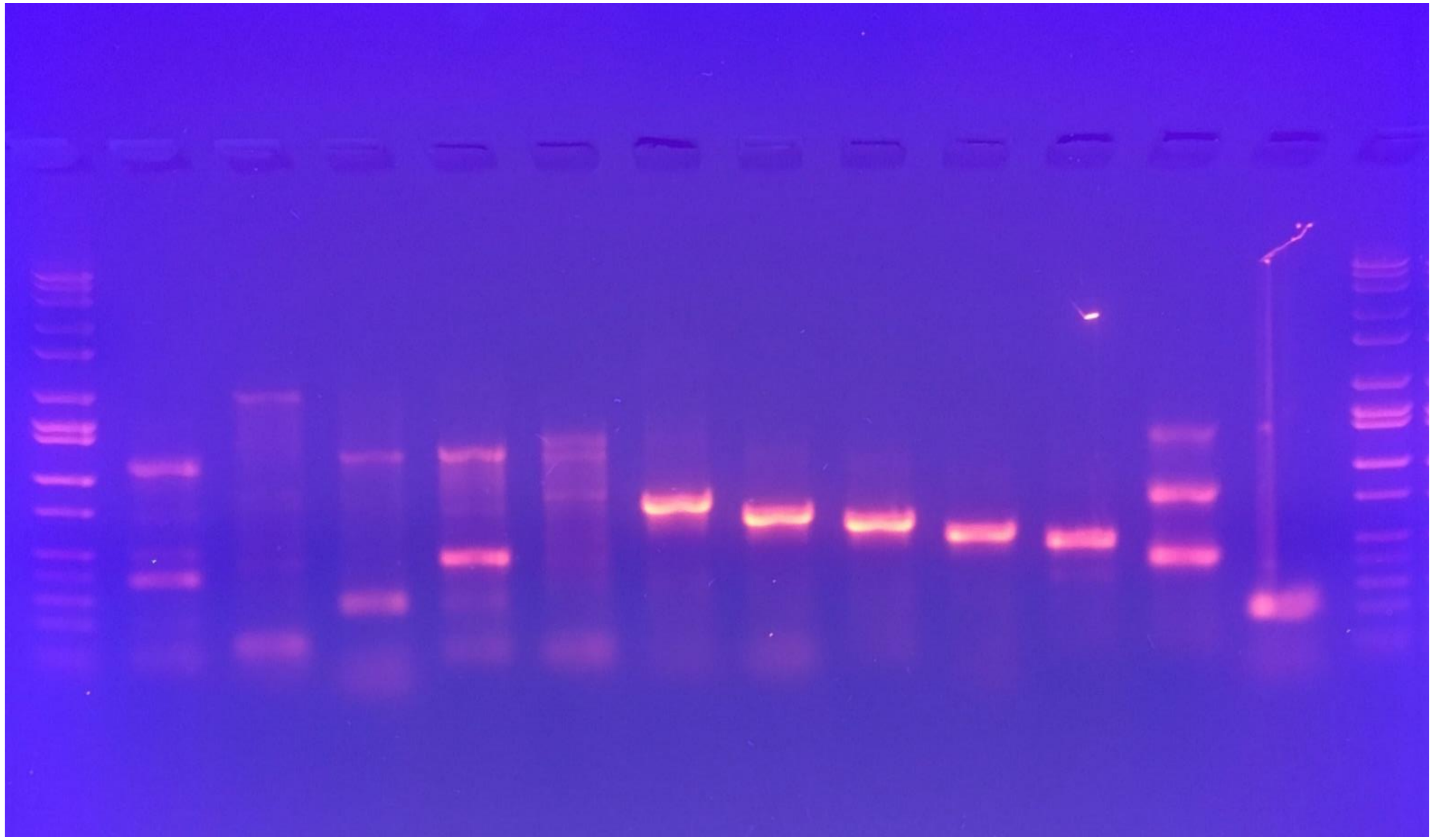

Fig 6, panel C

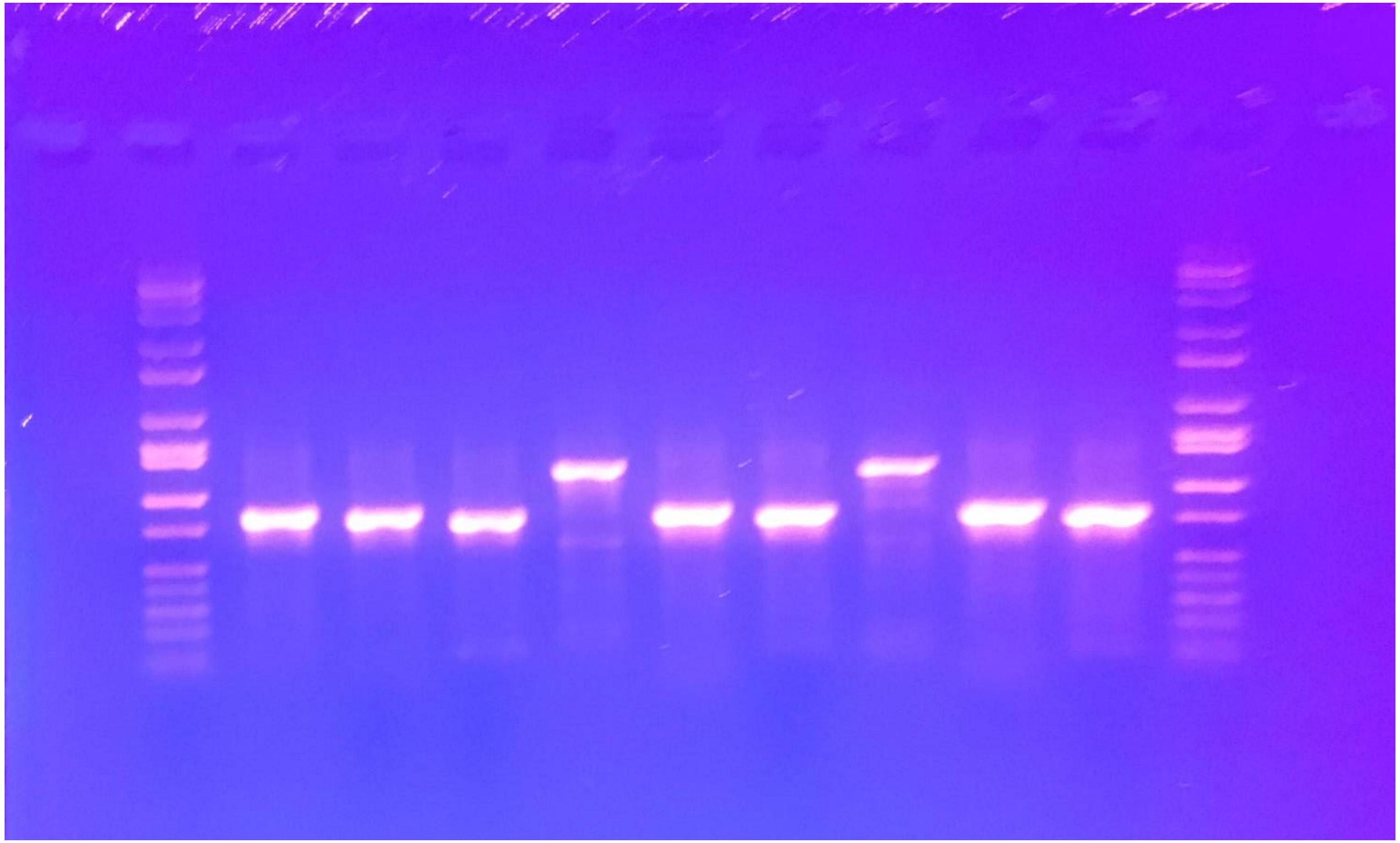

Fig 6, panel D

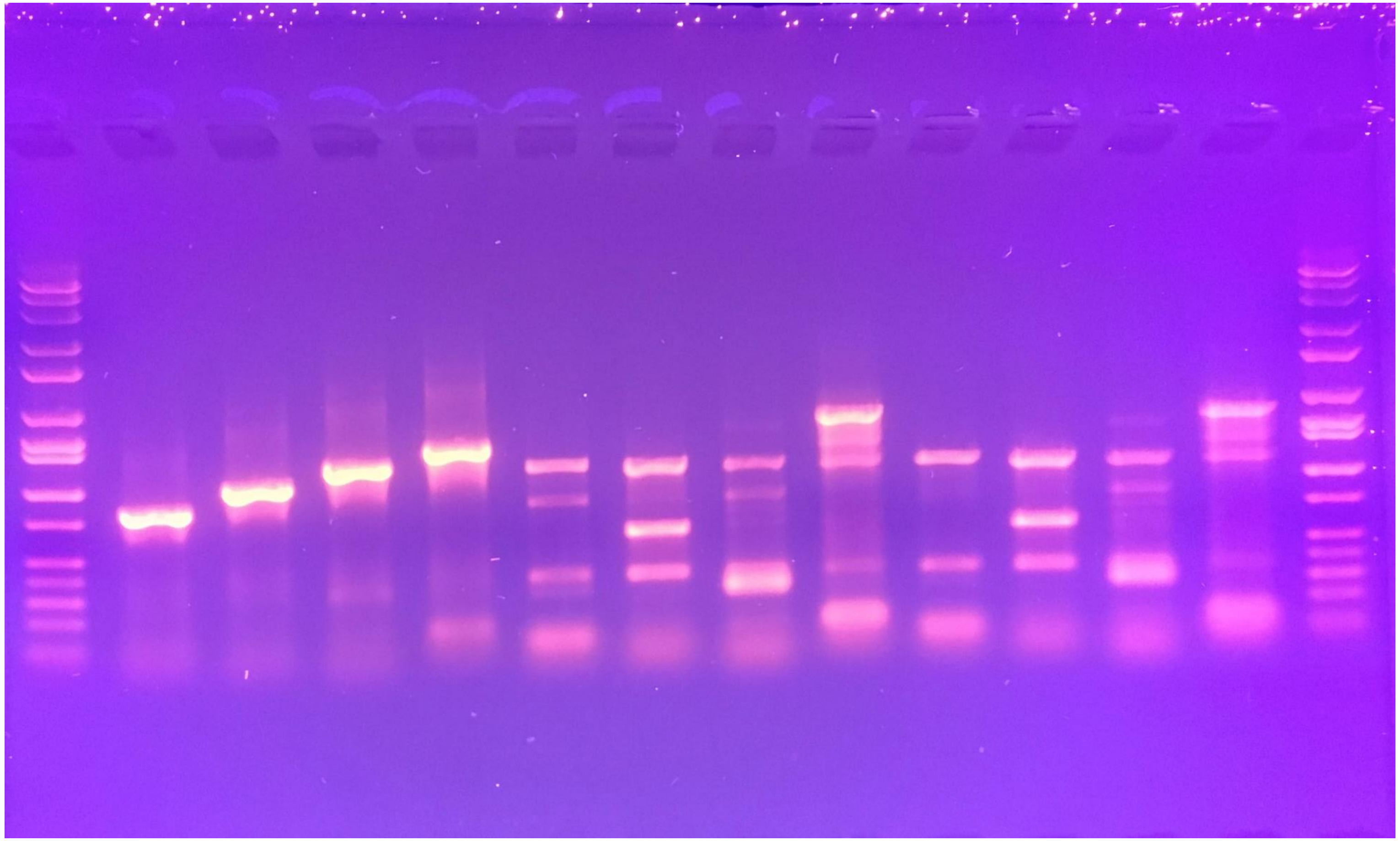

Fig 7, panel A

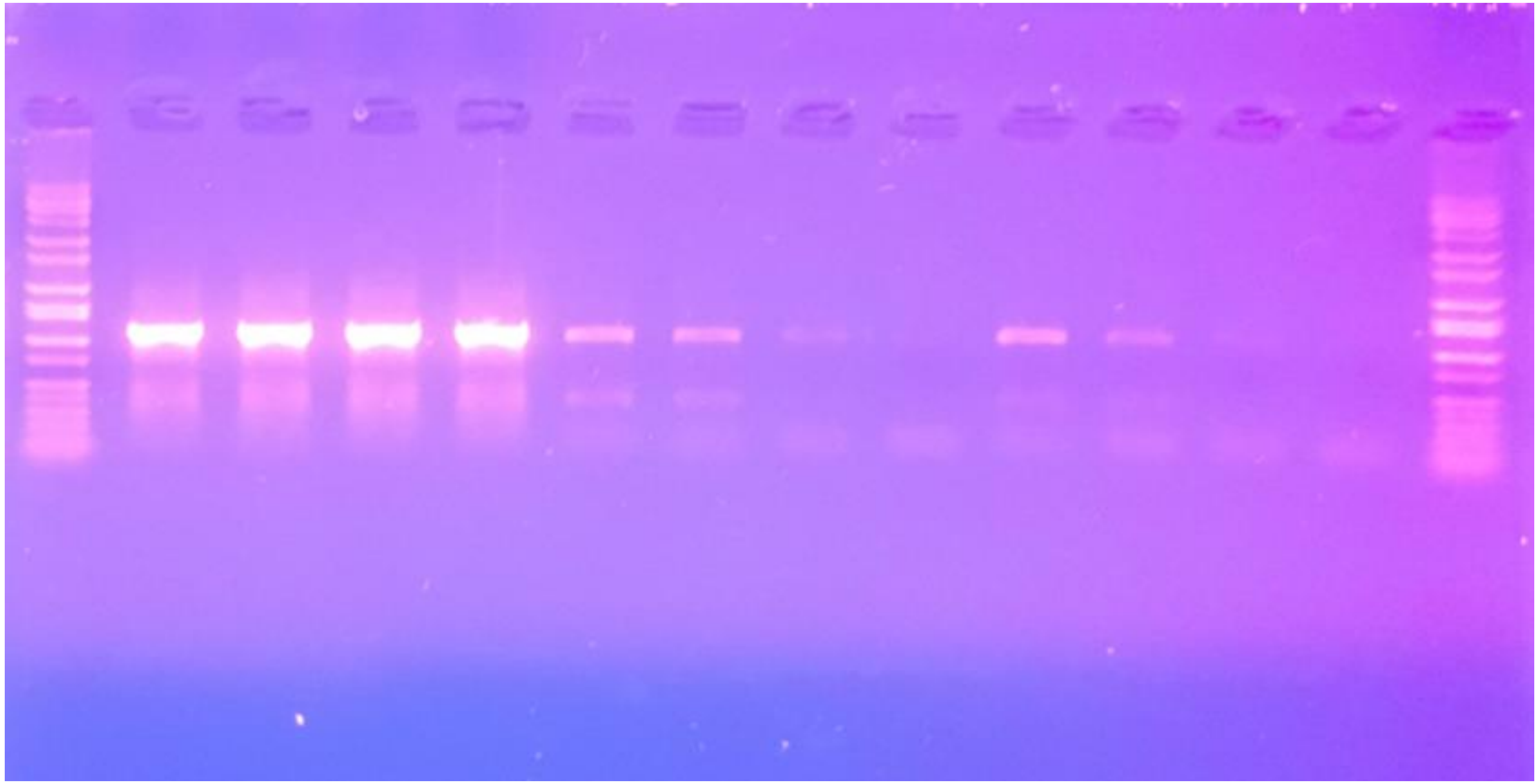

Fig 7, panel B

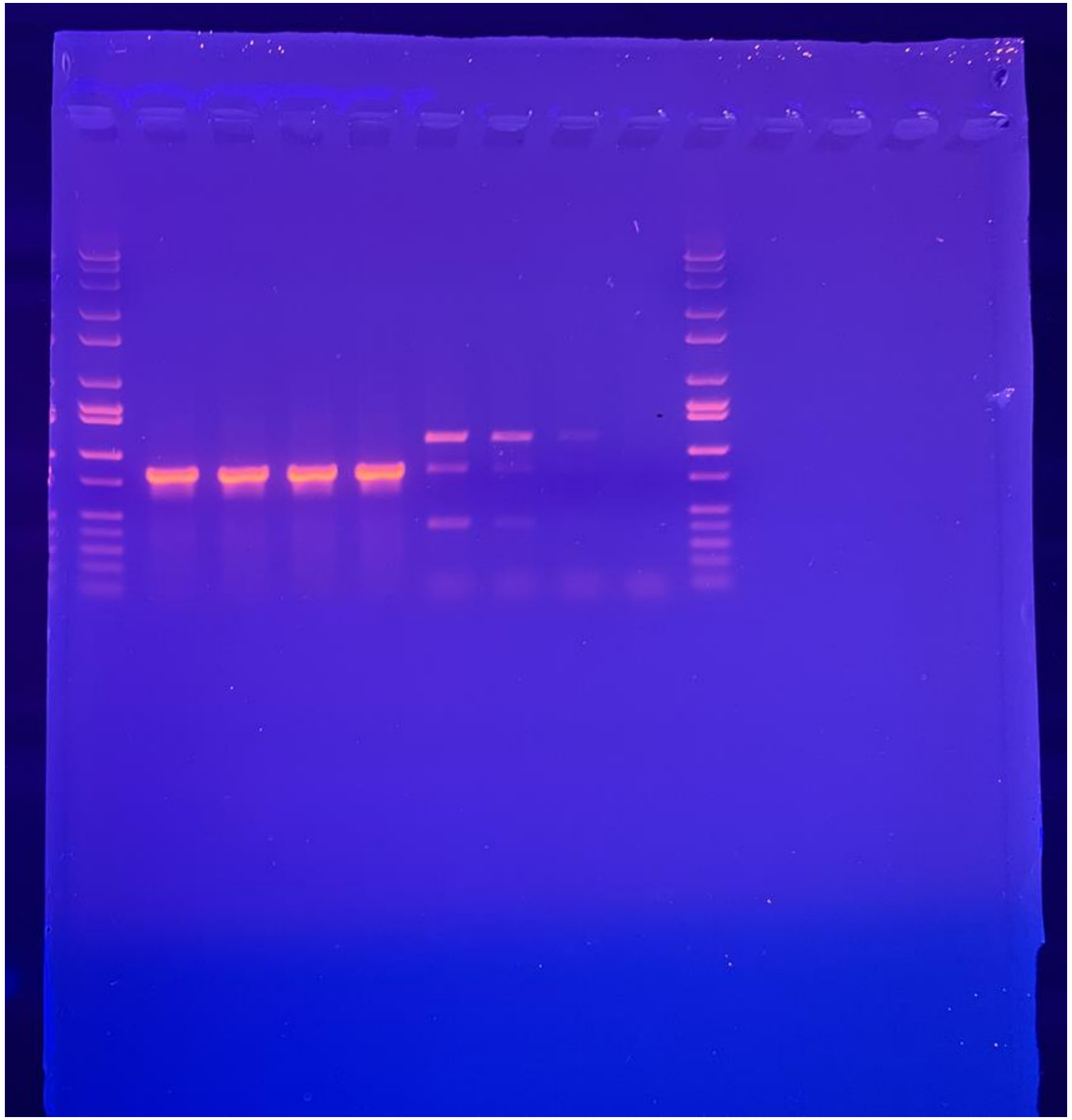

Fig 7, panel C

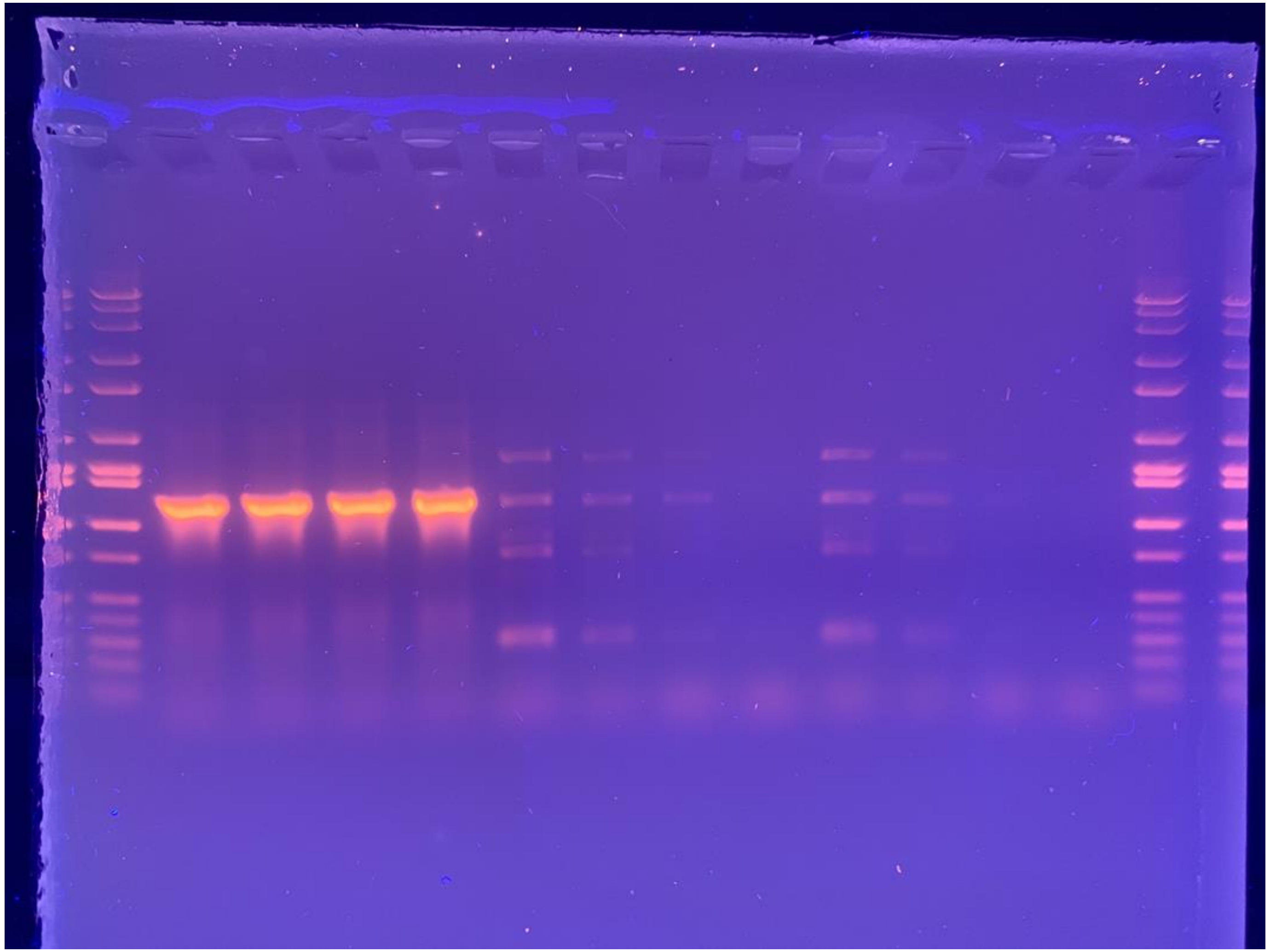

S6 Fig

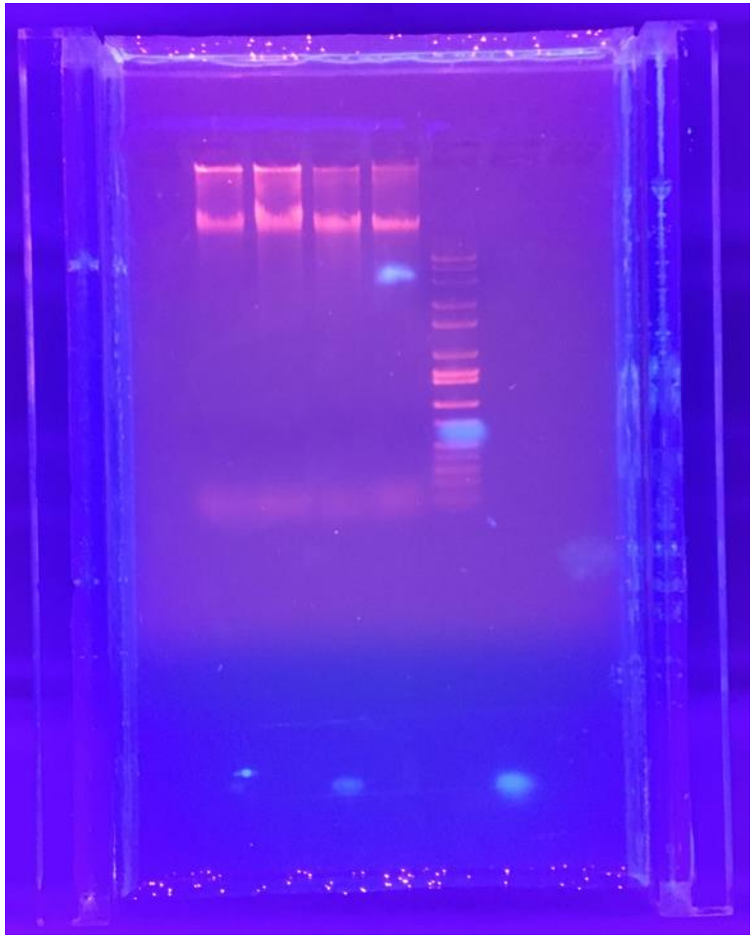

Supplement: S1 Raw Images — (PDF) [file pone.0230915.s009.pdf]
